# Targeting HDAC and proteostasis to eradicate quiescent cancer cells

**DOI:** 10.1038/s41417-026-01067-y

**Published:** 2026-08-13

**Authors:** Qi Jiang, Michelle Ertel, Austin Arrigo, Amjad Hijazzi, Sara Sannino, Jennifer L. Goeckeler-Fried, April Sagan, Ian Beddows, Betsy Ann Varghese, Daniel D. Brown, Wayne Stallaert, Adrian Lee, Amanda M. Clark, Hui Shen, Jeffrey L. Brodsky, Hatice U. Osmanbeyoglu, Ronald J. Buckanovich

**Affiliations:** 1 Obstetrics and Gynecology Magee-Womens Research Inst. & Foundation, Pittsburgh, PA USA; 2 School of Medicine, Tsinghua University, Beijing, China; 3 Division of Gynecologic Oncology, Department of Obstetrics and Gynecology, University of Pittsburgh, Pittsburgh, PA USA; 4 Department of Biological Sciences, University of Pittsburgh, Pittsburgh, PA USA; 5 Department of Biomedical Informatics, University of Pittsburgh, Pittsburgh, PA USA; 6 Van Andel Institute, Grand Rapids, MI USA; 7 Department of Computational and Systems Biology, UPMC Hillman Cancer Center, University of Pittsburgh, Pittsburgh, PA USA; 8 Department of Pharmacology, University of Pittsburgh, Pittsburgh, PA USA; 9 Department of Pathology, University of Pittsburgh, Pittsburgh, PA USA; 10 Department of Internal Medicine, UPMC Hillman Cancer Center, Pittsburgh, PA USA

**Keywords:** Cell biology, Cancer therapy

## Abstract

Chemotherapy remains the primary treatment for ovarian cancer (OvCa), and chemoresistance drives patient mortality. Cellular quiescence, reversible exit from the cell cycle, increases chemotherapy resistance as chemotherapies primarily target rapidly proliferating cells. Here, we report that CHD4 and MBD3, components of the nucleosome remodeling and deacetylase (NuRD) complex, are downregulated in quiescent OvCa cells (qOvCa). We find that either CHD4 or MBD3 knockdown or histone deacetylase inhibitors (HDACi), induce quiescence in OvCa cells. RNA-Seq and ATAC-seq analysis of HDACi-treated cells confirmed expression changes consistent with induction of quiescence. Additionally, HDACi-treated cells revealed downregulation of the RHO/RAC pathways. Suggesting downregulation of the RAC pathway could play a role in HDACi-mediated quiescence, RAC inhibitors (RACi) similarly induced quiescence. Both HDACi and RACi resulted in nuclear-to-cytoplasmic shifting of the pro-proliferative transcription factor MRTFA. which has been linked to quiescence. Further analysis of HDACi qOvCa indicated multiple alterations in proteostasis, including increased proteasome activity and autophagy. We find qOvCa cells are dependent on these pathways for survival, such that there is profound synergistic OvCa cell death with HDACi and proteasome- or autophagy-inhibitor combination therapy. Combined, this work supports HDACi as pharmacologic means to induce a quiescent state in OvCa cells and sensitize them to proteostasis-targeting drugs.

## Introduction

Worldwide, ovarian cancer (OvCa) remains the most lethal gynecological cancer, with a high mortality-to-incidence ratio. Approximately 324,400 new cases of OvCa were diagnosed worldwide in 2022 [1], and although most of these patients will have an initial response to surgery and chemotherapy, ~70% of patients will relapse and develop chemotherapy-resistant disease [2]. Understanding the mechanism of chemotherapy resistance and seeking methods to overcome resistance is essential to improve OvCa patient prognosis.

One important mechanism that contributes to chemotherapy resistance is cellular quiescence. Quiescent cells reversibly exit the cell cycle, stop proliferating, and are thus refractory to standard chemotherapies, which preferentially target rapidly proliferating cells [3, 4]. With the appropriate signals, quiescent cells can re-enter the cell cycle and drive cancer recurrence. A better definition of the factors that specifically regulate cancer cell quiescence could lead to targeted treatments to prevent chemotherapy resistance.

Our greatest understanding of quiescent cell biology comes from studies of yeast and adult stem cells. Quiescent cells demonstrate epigenetic changes and changes in chromatin remodeling [5–8], and these epigenetic changes are associated with reduced ribosome biosynthesis, altered RNA processing and protein translation [9, 10]. Furthermore, in *Saccharomyces cerevisiae*, the ubiquitin-proteasome system (UPS)—another key regulator of protein homeostasis (proteostasis)—appears to play an important role in quiescence; in one study, the proteasome assembly factor, Spg5, was found to be induced in quiescent cells and was essential for yeast survival [11].

While less well studied, our knowledge of cancer cell quiescence mirrors what has been observed in yeast and mammalian adult stem cells. In various cancer types, quiescent cancer cells have been described as a subset of cancer stem-like cells (CSCs) and are linked to chemoresistance [12–18]. Yet the contribution of quiescence in OvCa is ill-defined. However, experiments using patient-derived xenograft (PDX) models demonstrated that OvCa cells that survived chemotherapy were primarily Ki-67 negative and enriched for expression of the CSC markers ALDH and CD133 [19]. In addition, Gao et. al. identified a similar population of slow-cycling, CD24+, chemotherapy-resistant OvCa cells that drive cancer recurrence in primary human tumors [20]. We recently reported that the NFATC4 transcription factor is activated by chemotherapy and can drive a chemotherapy-resistant quiescent cell state [21]. Furthermore, we found that, in response to chemotherapy, quiescent cells secrete follistatin, which inhibits growth but triggers chemotherapy resistance in neighboring cells. As such, follistatin knockout increased chemosensitivity and reduced chemotherapy resistance both in vitro and in vivo.

To better define the mechanisms that initiate and maintain quiescent OvCa (qOvCa) cells, we recently performed single-cell RNA-sequencing analysis on isolated primary qOvCa cells [22]. We found that many qOvCa differentially expressed genes are transcriptional targets of the Myocardin-Related Transcription Factor-A (MRTFA)/Serum Response Factor (SRF) pathway, and inhibition of MRTFA induces quiescence in OvCa [22]. Besides, MBD3 and CHD4, two major components of the epigenetic regulatory nucleosome remodeling and deacetylase (NuRD) complex, are known SRF targets that were found to be downregulated in qOvCa. While the NuRD complex plays an important role in regulating cell cycle regulators and transcription factors [23, 24] and has been linked to tumorigenesis, its role in quiescence has been relatively unexplored. In this study, we find that inhibition of NuRD complex activity—either by knockdown of MBD3/CHD4 or inhibition of NuRD complex histone deacetylase activity with histone deacetylase inhibitors (HDACi) is associated with reduced MRTFA, alters expression of numerous cell cycle regulators and drives OvCa cells into quiescent state. Furthermore, both primary quiescence and HDACi-induced quiescence were associated with altered proteostasis, including upregulation of proteasome activity and autophagy-associated proteins, HDACi-induced quiescent cells are sensitized to proteasome and autophagy inhibitors. Furthermore, to better define a universal quiescent cancer cell signature, we overlaid multiple qOvCa RNA-Seq datasets with essential quiescence genes identified in yeast. As a result of this effort, we report on a new core quiescence signature that is reflected across multiple tumor types.

## Results

### The NuRD complex components MBD3 and CHD4 are downregulated in quiescent OvCa

**Fig. 1:**
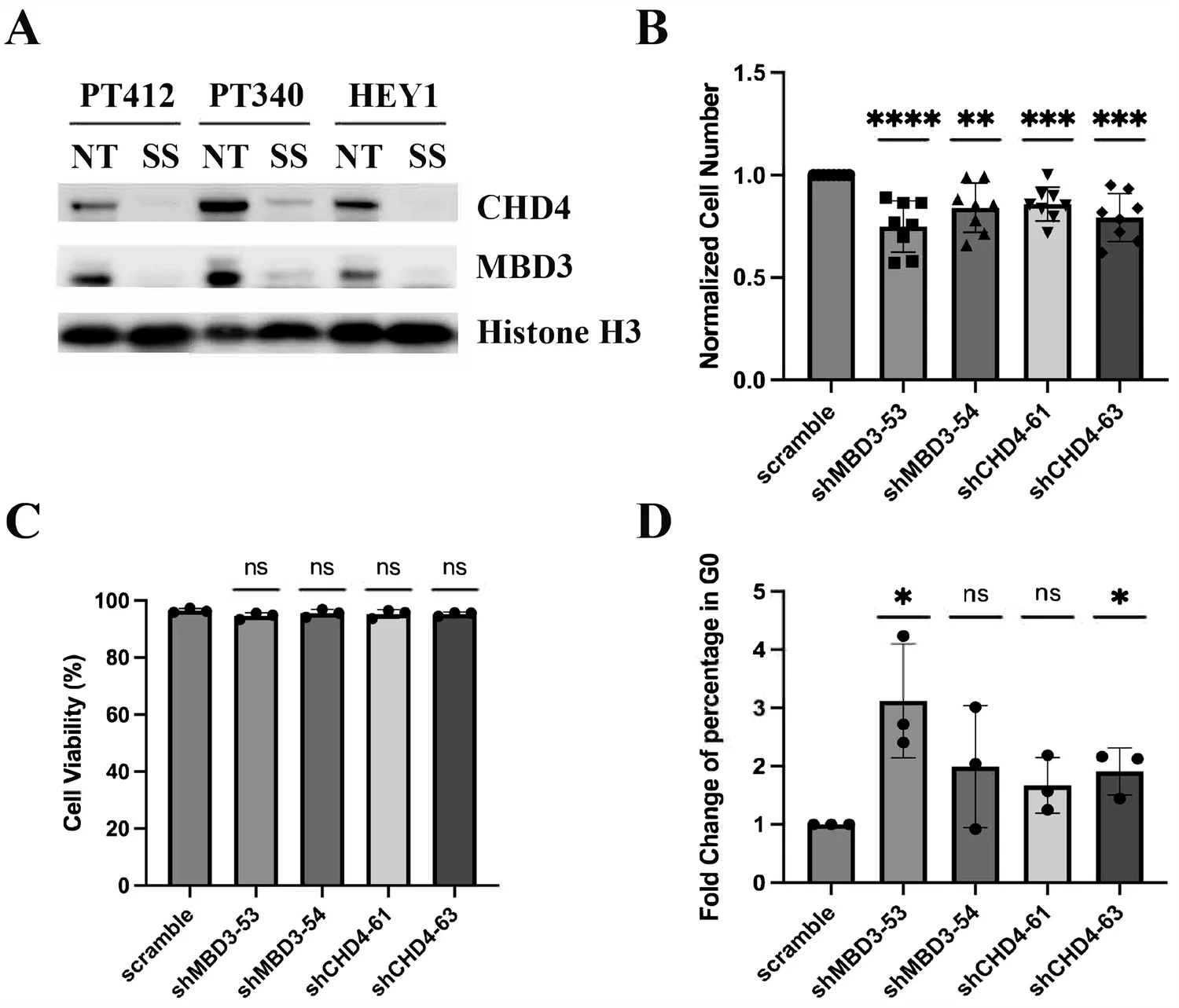
Knockdown of MBD3/CHD4 drives quiescence in OvCa cells. **A** Western blot analysis of MBD3 and CHD4 in control and cells that were serum starved for 72 h to induce quiescence in PT412, PT340, and Hey1 cell lines. **B** Cell numbers in PT412 cells 72 h after transfection with scrambled control, MBD3, or CHD4 shRNAs. Individual replicates from three experiments are shown. **C** Cell viability of PT412 cells transfected (as above) with scrambled, MBD3, and CHD4 shRNA and assessed via PI/Annexin-V apoptosis assay. **D** Summary of cells determined to be in G0, using flow cytometry of Fucci reporter (CDT1(30/120)-mcherry/p27K^-^-mVenus)-labeled Hey1 cell line treated with scrambled control, MBD3, and CHD4 shRNA. Cell viability and G0 analysis represent means from replicate experiments in triplicate, with *p* values calculated using two-tailed Student’s *t* tests, comparing means between groups.

To identify primary quiescent ovarian cancer cells, we recently performed single-cell RNA sequencing of primary qOvCa cell lines (PT340 and PT412) [22]. ALDH and CD133-positive ovarian cancer cells were isolated by FACS and seeded individually in a microfluidic chip. After culturing for 5 days, 100 quiescent (single cell in well) and 200 proliferating ovarian cancer cells were retrieved and collected for CellSeq2 single-cell RNA-sequencing [25]. This analysis indicated qOvCa cells had downregulated mRNAs corresponding to two components of the NuRD complex, MBD3 and CHD4 [26]. Western blot analysis in control OvCa cells and serum starvation-driven qOvCa cells confirmed the depletion of the MBD3 and CHD4 proteins in three OvCa cell lines (PT340, PT412 and HEY1) (Fig. 1A). To determine whether MBD3 and CHD4 downregulation was a result or potential driver of quiescence, we next performed siRNA and shRNA knockdown studies. Knockdown efficiency of shRNA (Supplementary Fig. S1A, B) and siRNA (Supplementary Fig. S1C, D) were validated by RT-qPCR and Western blot. Consistent with increased quiescence, knockdown of either MBD3 or CHD4 by shRNA resulted in decreased cell proliferation (Fig. 1B) with no change in cell viability as measured by an apoptosis assay in PT412 cells (Fig. 1C). Similar results were shown in siRNA-induced MBD3 or CHD4 knockdown in PT412 cells (Supplementary Fig. S1E, S1F). Furthermore, cell cycle analysis using the Fucci fluorescent cell cycle reporter system [27] in HEY1 cells demonstrated that MBD3 and CHD4 knockdown were associated with increased p27Kip/CDT1-positive cells; i.e., cells in the G0 phase of the cell cycle (Fig. 1D). These findings imply that decreased MBD3 and/or CHD4 expression induces OvCa quiescence.

### HDAC inhibitors induce a quiescent state in OvCa cells

**Fig. 2:**
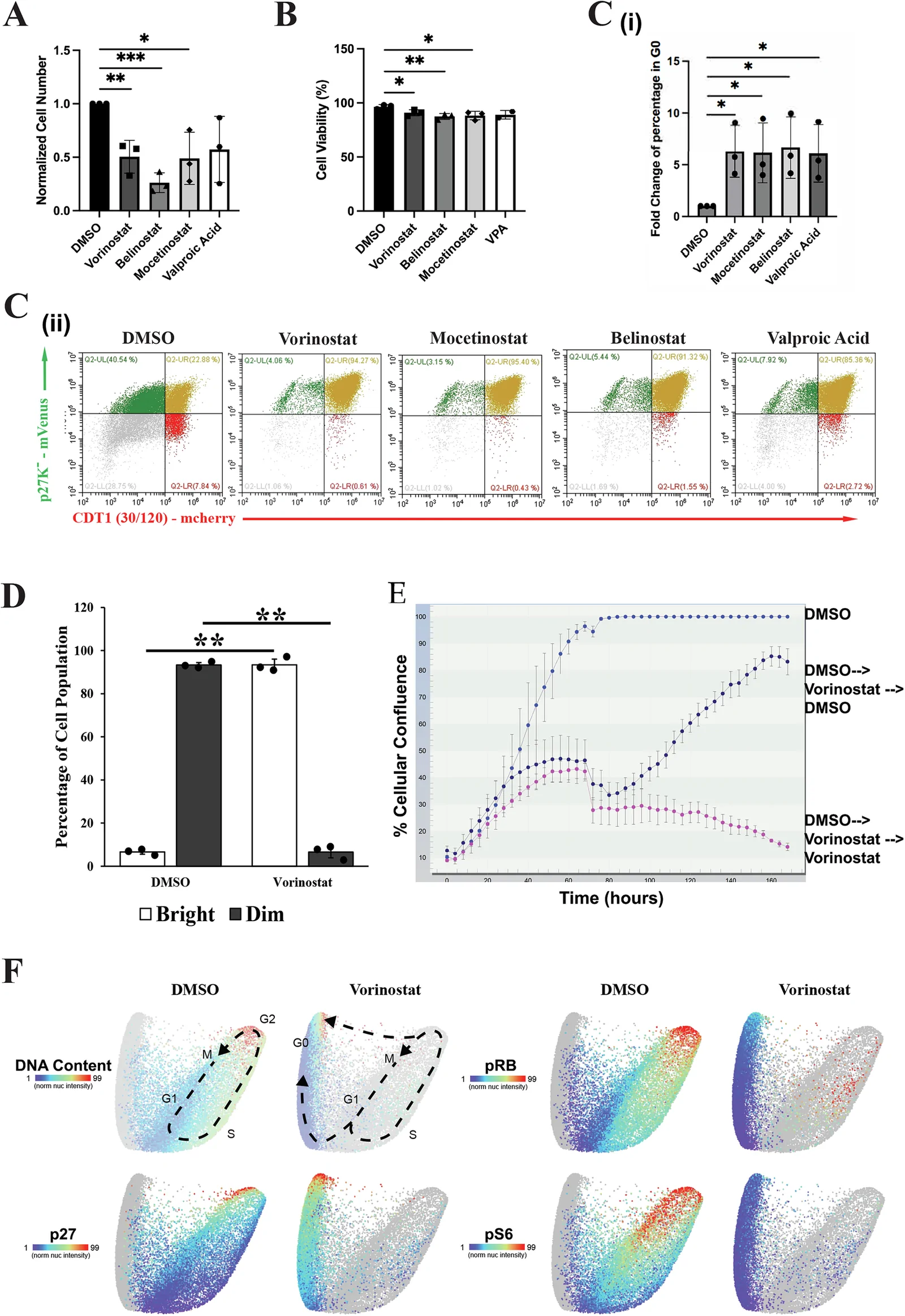
Pharmacologic inhibition of NuRD complex induces quiescence in OvCa cells. Normalized cell number (**A**) and viability (**B**) in cells treated for 48 h with DMSO, vorinostat, mocetinostat, belinostat, or VPA for the indicated OvCa cell lines (PT412, PT340, Hey1, each). Each point represents average results from an individual cell line. **C** (i) Summary and (ii) representative flow cytometry plots of Fucci reporter (CDT1(30/120)-mcherry/p27K^−^-mVenus)-labeled Hey1 cell line treated with DMSO, vorinostat, mocetinostat, and belinostat for 48 h. Cells in G0 state express both p27K-mVenus and CDT1(30/120). **D** Summary of flow cytometry assessment of cell trace violet (CTV)-labeled retention/dilution of PT412 cells treated with or without vorinostat for 5 days. Percentage of Dim (proliferating) or Bright (quiescent) cells in total cell population. **E**. Real-time cell proliferation analysis of Hey1 cells treated with (1) DMSO, (2) vorinostat 72 h followed by DMSO (vorinostat→DMSO), (3) vorinostat. **F** Cell cycle map of DMSO- and vorinostat-treated PT340 cells. (Dotted lines indicate proliferative and arrest trajectories.). Each experiment was independently repeated three times, with *p* values calculated using two-tailed Student’s *t* tests, comparing means between groups.

Because the redundancy among MBD/CHD family members may reduce the impact of knockdown, we next evaluated whether pharmacologic inhibition of the HDACs, which are also components of NuRD complex using HDAC inhibitors (HDACi) induces quiescence. We treated OvCa cells with the pan-HDAC inhibitors vorinostat (SAHA), belinostat, or valproic acid (VPA), or the HDAC1, 2, 3, 11 selective inhibitor mocetinostat for 48 h. Consistent with the induction of quiescence, HDACi-treated cells showed a significant reduction in cell numbers with minimal impact on cell death (Fig. 2A, B). Cell cycle analysis using the HEY1-Fucci cell cycle reporters (p27-mVenus and CDT1-mCherry) suggested each of the HDACi also induce a quiescent state, with >90% of cells arrested in the G0 phase of the cell cycle (Fig. 2C). To confirm this finding, vital dye (cell trace violet-CTV) was used to label OvCa cells and track cellular proliferation. The cells were then treated with vorinostat. Flow cytometry demonstrated that vorinostat treatment significantly increased the percentage of CTV-bright (i.e., non-dividing) cells compared to DMSO control cells in PT412 (Fig. 2D). To determine whether the vorinostat-induced cell cycle arrest is reversible, we performed real-time imaging of OvCa cells. Replicated groups of cells were cultured for 24 h, allowing cells to reach linear growth, then cell confluency was recorded, and cells were treated with (i) DMSO, (ii) vorinostat continuously, or (iii) vorinostat for 72 h before vorinostat was washed out and DMSO re-applied. Indicating reversibility of cellular arrest, vorinostat washout allowed cells to resume proliferation within ~24 h in multiple OvCa cell lines (HEY1, PT412, Kuramochi) (Fig. 2E, Supplementary Fig. S2).

To further confirm induction of quiescence, we used multispectral iterative immunofluorescence to evaluate expression of 28 cell cycle effectors, including CDKs, cyclins, p21, p27, phospho-Rb, and DNA content in DMSO control and vorinostat-treated OvCa cells. The intensity of phospho-Rb, p27, and other cell cycle regulators in DMSO-treated PT340 cells was then used to define a cell cycle map, as previously described [28–32]. Vorinostat-treated cells demonstrated a staining pattern, with most cells in G0 and pre-G0 state, which included a reduction in nuclear p27, pRB, and pS6 (Fig. 2F, Supplementary Fig. S3). Together, these data indicate that either genetic downregulation of CHD4 or MBD3 or pharmacologic inhibition by HDAC inhibitors, which reduced the activity of the NuRD complex, induces OvCa cellular quiescence, reflecting the potential role of NuRD complex in regulating quiescence.

### Vorinostat-induced quiescent cells and primary quiescent cells exhibit similar transcriptional profiles

**Fig. 3:**
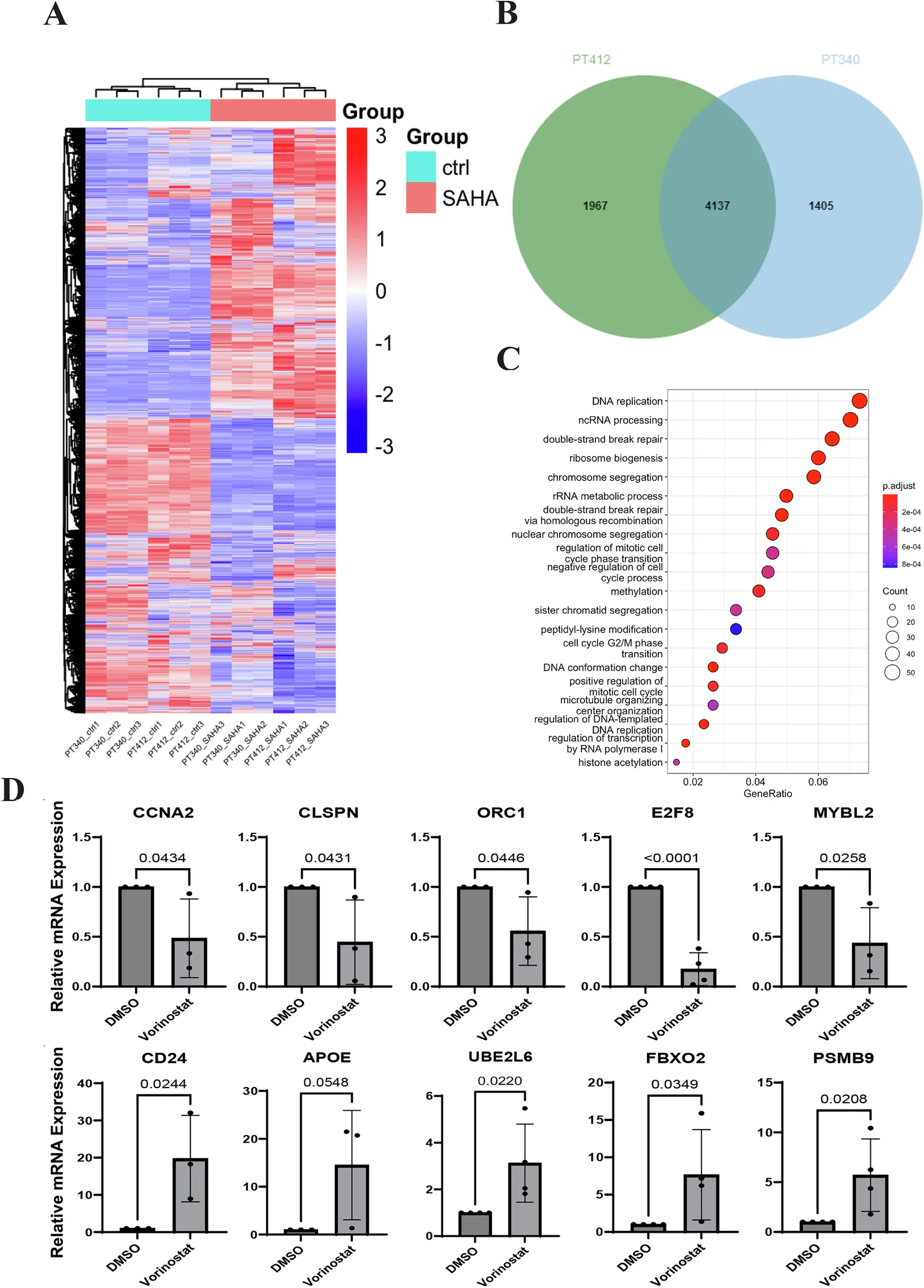
RNA sequencing of vorinostat-treated cells shows characteristics of quiescent cells. **A** Heatmap of RNA-seq of top 15,000 genes in control and vorinostat-treated cells in PT340 and PT412. **B** Venn diagram showing numbers of overlapped DEGs in vorinostat-treated PT340 and PT412. **C** GO biological process analysis for genes downregulated (LFC < 1.0 and *p*.adj < 0.05) in vorinostat-treated PT340 and PT412 cells, showing top 20 pathways. **D** RT-qPCR evaluation of expression of the indicated genes in control and vorinostat-treated OvCa cell lines (PT340, PT412, A2780), with each data point representing an average of three technical replicates for a given cell line.

To examine the transcription changes associated with vorinostat-induced quiescence in OvCa cells, we performed bulk RNA sequencing. Two OvCa cell lines (PT340, PT412) were treated with DMSO or vorinostat for 48 h, and total RNA was extracted and used for bulk RNA sequencing (*n* = 3/treatment group). A principal component analysis (PCA) plot showed consistent effects amongst triplicate aliquots of control samples and treated cells (Supplementary Fig. S4). A heatmap of the top 15,000 expressed genes also showed samples clustered by treatment instead of cell line identity (Fig. 3A). An analysis (log fold change (LFC) > 1 or < −1, *p*-adjust <0.05) of differentially expressed genes (DEG) revealed 5543 DEGs in PT340 and 6105 DEGs in PT412 cells, with 4137 DEGs overlapping in the two cell lines (Fig. 3B) [33].

Next, we performed Gene ontology (GO) biological process analysis and discovered overlapped downregulated DEGs consistent with altered ribosome biogenesis, RNA processing, DNA replication, cell cycle phase transition, cell division, and DNA conformation, all of which are consistent with the characteristics of quiescent cells (Fig. 3C) [34]. In addition to expression changes in numerous cell cycle-related genes, ubiquitin-proteasomal system (UPS)-related genes were also differentially expressed in vorinostat-treated cells, as compared to controls, across both cell lines. Specifically, RT-qPCR analysis of cell cycle factors (CCNA2, CLSPN, E2F8, MYBL2, ORC1), the cancer progression marker APOE [35], the cancer stem cell marker CD24 [36, 37], and UPS factors (UBE2L6, FBXO2, PSMB9) confirmed RNA-Seq data (Fig. 3D). These results are also consistent with iterative immunofluorescent cell cycle mapping, which showed CCNA2, CCNB2, and CCND1 to be downregulated at the protein level (Supplementary Fig. S3). Combined, these data indicate that vorinostat treatment induces quiescence in OvCa cells.

### HDACi modulates RHO GTPase-associated pathways and MRTF-A localization

**Fig. 4:**
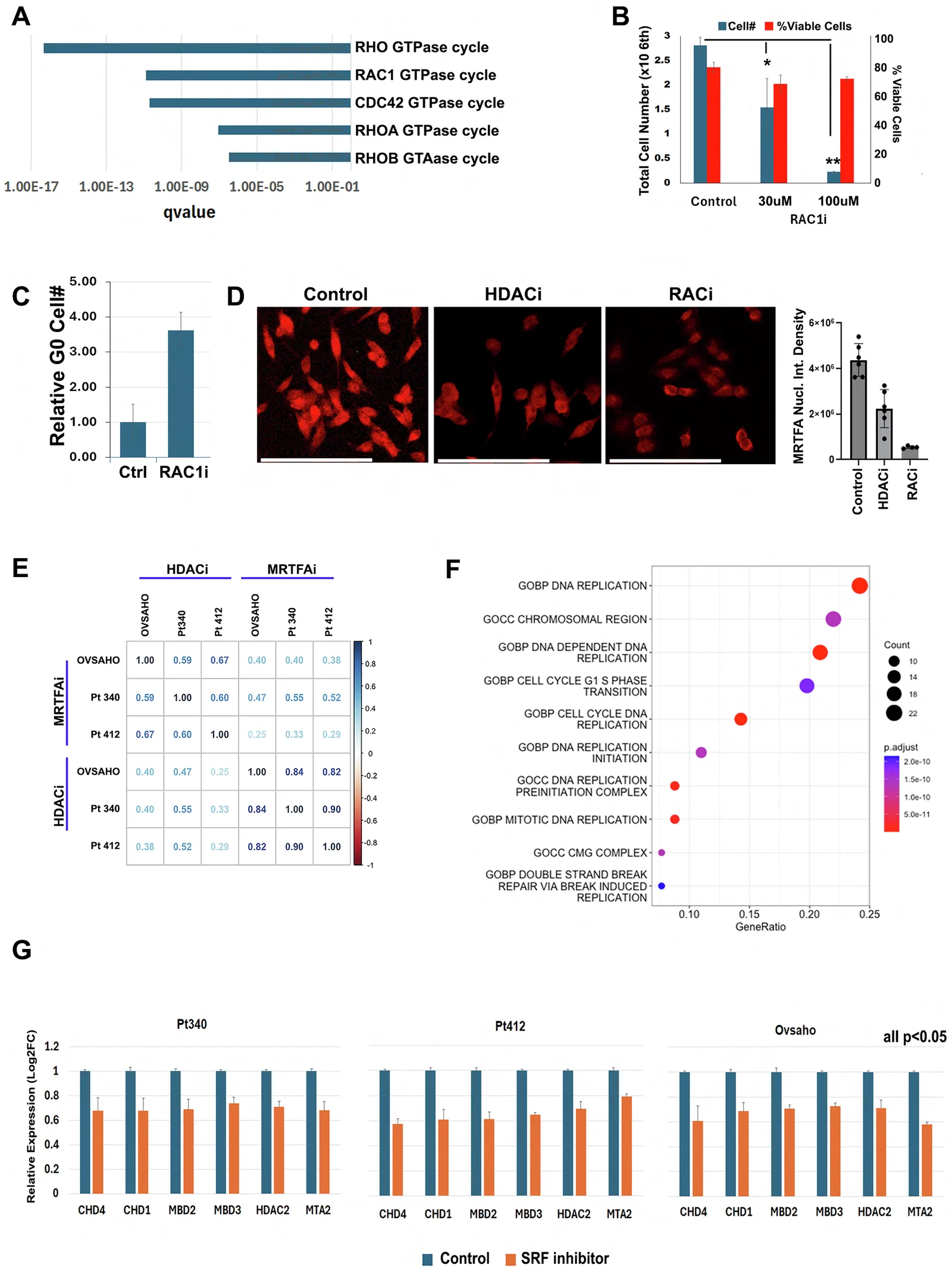
HDACi treatment results in RACi-mediated inhibition of the MRTFA/SRF pathway. **A** ATAC-q gene ontology reactome enrichment of genes with closed chromatin following HDACi (vorinostat (SAHA)) treatment. **B** Total cell number and percentage of viable cells in Pt340 cells treated with the indicated doses of the RACi inhibitor NSC 23766. **C** Relative proportion of PT412 cells in G0 (assessed using FUCCI reporters) in control and RACi-treated cells. **D** Representative MRTFA immunofluorescence and quantification of nuclear MRTFA stain in control, HDACi (SAHA)- and RACi-treated pt 340 cells. **E** Pearson correlation coefficient of RNA-Seq-based gene expression for the indicated cell lines and drug treatments, showing strong correlation for SAHA and MRTFA inhibitor (10 µM CCG257081) treatment. **F** GO biologic process gene set enrichment analysis of genes downregulated by both HDACi and MRTFAi (LFC < −1 with *p* < 0.05 in at least 2 cell lines). **G** Relative gene expression for the indicated NuRD components after treatment with the MRTFAi.

To better define the mechanisms of HDACi-induced quiescence, we performed ATAC-seq on control and HDACi-treated cells PT340, PT412, and OVCAR8. Pathway enrichment analysis of the genes associated with closed chromatin after HDACi administration revealed a profound enrichment for genes encoding proteins associated with the RHO-GTPase, including the RAC1 GTPase cycle, CDC42 GTPase cycle, RHOA GTPase cycle, and RHOB GTPase cycle (Fig. 4A). To assess whether inhibition of these pathways in turn contributes to HDACi-induced quiescence, we treated PT340 cells with a selective RAC1 inhibitor (RAC1i; NSC 23766), a CDC42 inhibitor (CDC42i; ML-141), and a RHOA inhibitor (RHOAi; Rhosin hydrochloride) and evaluated the impact on total cell number and viability. The RhoA inhibitor had a minimal impact on growth and viability, while the CDC42 inhibitor proved generally toxic, with reduced cell numbers and cellular viability (Supplementary Fig. S5). However, treatment with the RAC1 inhibitor significantly reduced cell numbers without significantly impacting cell viability in PT340 (Fig. 4B). To confirm that RAC1 inhibition induces quiescence, we treated PT412-FUCCI cell cycle reporter-labeled cells with the RAC1i and observed a ~3-fold increase in cells in G0 (Fig. 4C).

Beyond quiescence, the RHO/RAC GTPase family of proteins also regulates the MRTFA/SRF pathway, a critical driver of cellular migration and proliferation [38, 39]. We recently reported that forced cytoplasmic localization/inhibition of MRTFA can induce a quiescent phenotype in OvCa cells [22]. Therefore, we assessed the impact of HDACi or RAC1i on loss of MRTFA nuclear localization and, thus, inhibition of the MRTFA/SRF pathway and quiescence induction. Indeed, using immunofluorescence localization of MRTFA, we found that treatment with either HDACi or RAC1i was associated with loss of nuclear MRTFA in PT340 (Fig. 4D).

We recently reported that the MRTFA/SRF inhibitor CCG257-081 [40], which induces nuclear to cytoplasmic translocation of MRTFA with subsequent inhibition of MRTFA/SRF transcriptional activity, can drive quiescence in ovarian cancer cells [22]. We next compared the RNA-Seq profile of HDACi-treated cells with the recently reported RNA-Seq profile of CCG257-081-treated cells [22]. Within a given cell line, HDACi- and MRTFAi-treated cells shared a significant overlap (Fig. 4E). Once again, consistent with each inhibitor inducing quiescence, gene set enrichment analysis of downregulated genes (−1 log fold change) for CCG257-081 and HDACi showed downregulation of DNA replication and cell cycle-related pathways (Fig. 4F). Downregulated genes included CCNA2, ORC2, BRCA2, and MCM4, among others. Interestingly, MRTFA inhibitor treatment was associated with downregulation of several NuRD complex components, suggesting a potential feed-forward suppression cycle (Fig. 4G).

### Identification of a core quiescence signature

**Fig. 5:**
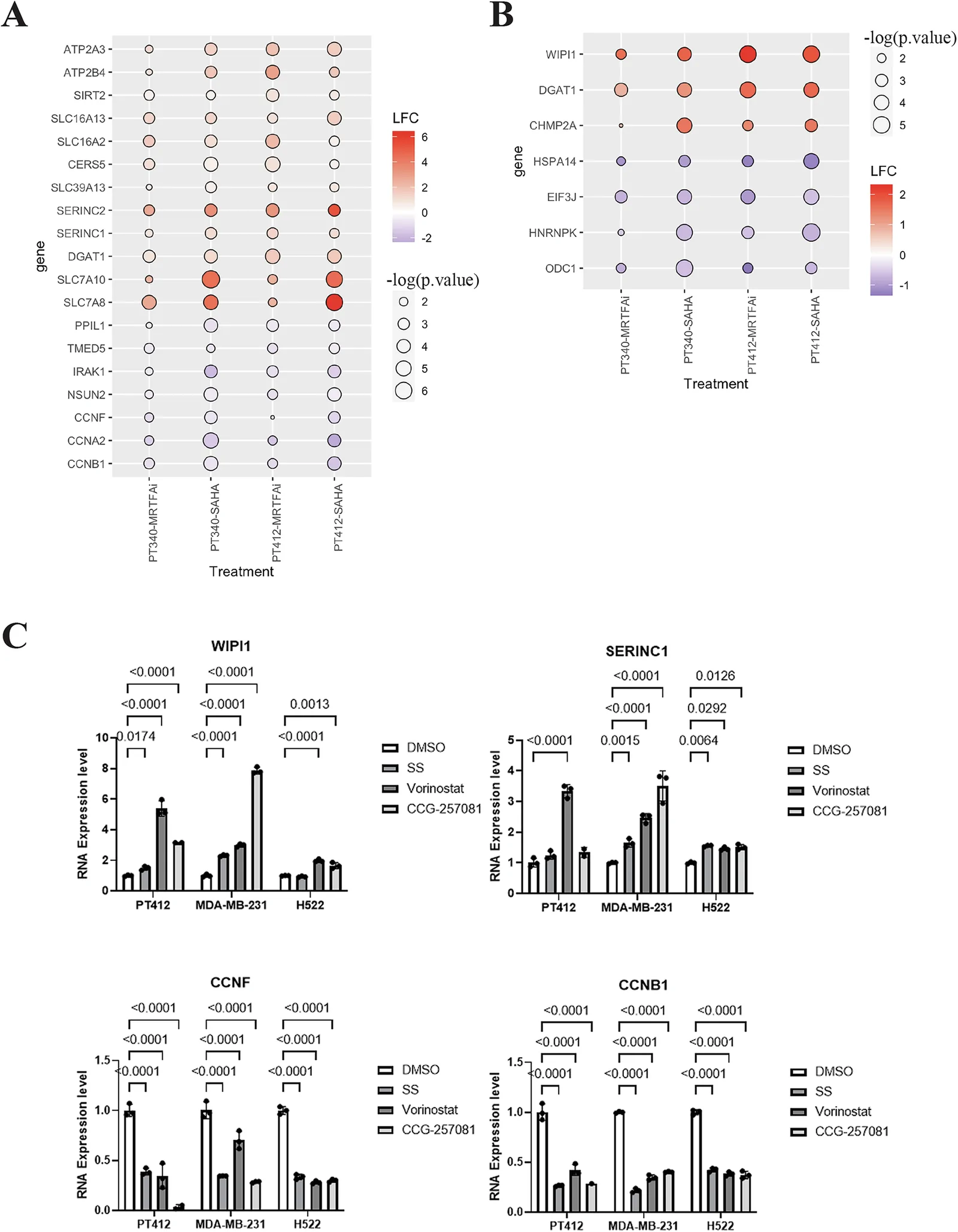
Core quiescence signature shared by *Schizosaccharomyces* and OvCa cells. **A** Heat map of quiescence-essential genes in *Schizosaccharomyces* and differentially expressed genes from ovarian cancer cells treated with quiescence-inducing drugs (vorinostat and MRTFA inhibitor CCG081). Cutoffs in qOvCa cells are LFC > 0.5 or <−0.5, *p* value < 0.05; Cutoff in *Schizosaccharomyces*: Quiescent effect > 1, *p* value < 0.05. **B** Heat map of *Schizosaccharomyces* mitotic competence-essential genes altered in differentially expressed genes from ovarian cancer cells treated with quiescence-inducing drugs. (Cutoffs in qOvCa cells are LFC > 0.5 or <−0.5, *p* value < 0.05). **C** Validation of quiescence core gene alternation in serum starvation-, vorinostat-, and CCG-257081-induced quiescence in PT412, MDA-MB-231, and H522 cell lines.

To better define the changes in gene expression and the identification of putative therapeutic targets in quiescent cells, we used existing data sets to identify a universal cancer “quiescent cell core signature”. To this end, we compared our existing RNA-Seq datasets with two different quiescent cell RNA signatures identified in *Schizosaccharomyces*, a well-established model of quiescence; Klosinska et al. reported the transcriptional profiles of nutrient depletion-induced quiescence [41], and Sajiki et al. reported genes essential for the mitotic competence for *Schizosaccharomyces* cells to exit quiescence [42]. We next overlaid these datasets with human orthologs identified after RNA sequencing of (i) vorinostat-induced quiescent cells, and (ii) the recently reported MRTFA inhibitor-induced quiescent cell signature [22]. Based on our analysis, we identified 25 genes, including upregulated genes (e.g., SLC7A8, WIPI1, and SERINC2) and downregulated genes (e.g., CCNF, CCNB1, HSPA14, and IRAK1), which we suggest represents a conserved quiescent cell core signature (Fig. 5A, B). To validate the core signature across cancer types, we performed RT-qPCR in DMSO-, serum starved-, vorinostat-, and CCG-257081-treated OvCa, lung cancer, and breast cancer cells (PT412, MDA-MB-231, H522). Consistent with the identification of a core signature, SERINC1, WIPI1, CCNF, CCNB1, and CCNA2 were similarly regulated across cancer types under different treatments (Fig. 5C).

### Quiescent OvCa cells are sensitive to proteostasis pathways

**Fig. 6:**
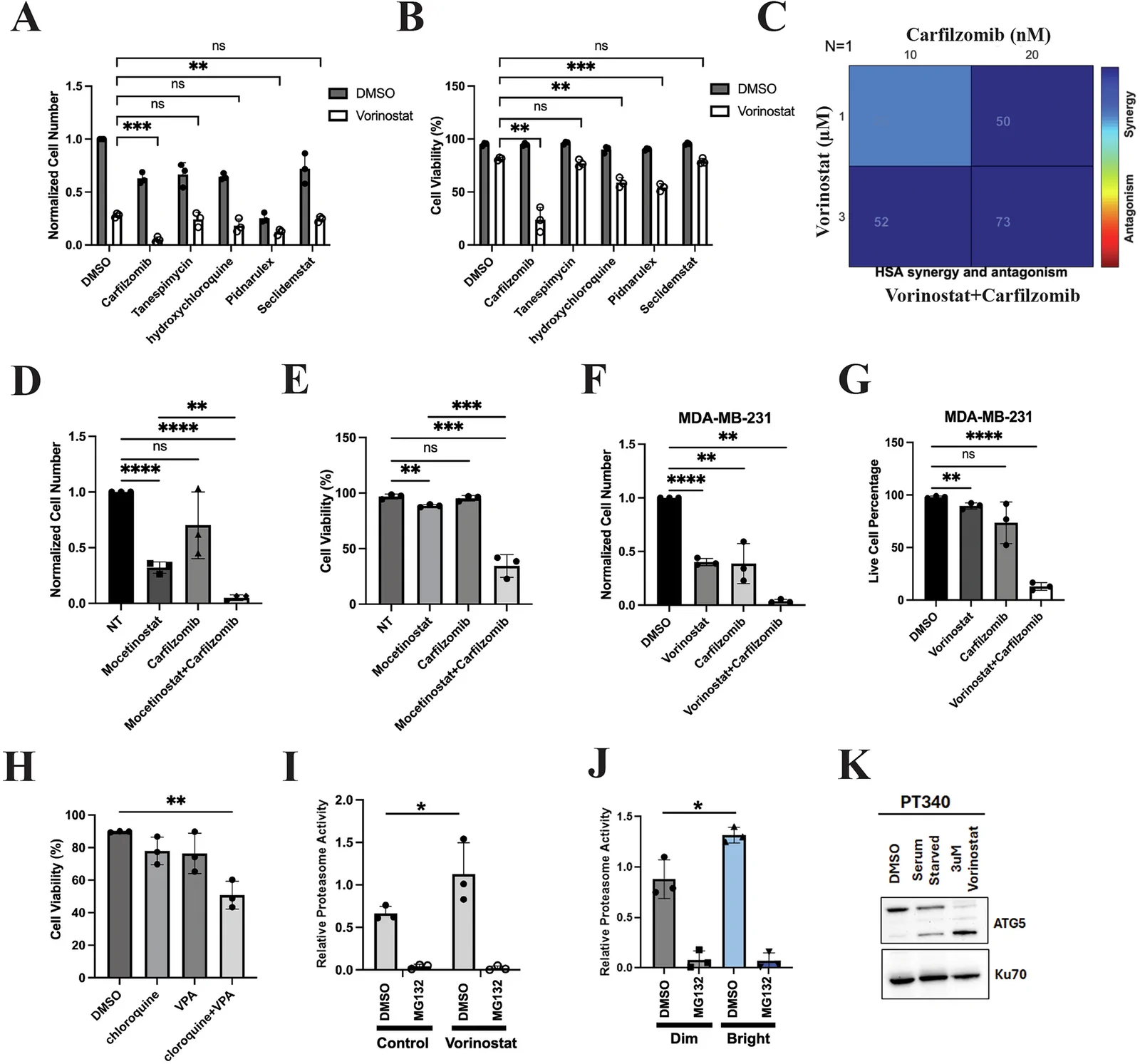
qOvCa cells have altered proteostasis, and targeting proteasome induces quiescent cancer cell death. **A**, **B** Normalized cell number and viability of PT412 cells treated with DMSO or vorinostat (SAHA) and the indicated compounds for 48 h. **C** HSA synergy heat map for vorinostat and carfilzomib. **D** Normalized cell number of DMSO, mocetinostat, carfilzomib single-drug treatment, and combination treatment for 48 h in PT340. **E** Cell viability of DMSO, mocetinostat, carfilzomib single-drug treatment, and combination treatment for 48 h in PT412. **F** Normalized cell number of DMSO, vorinostat, carfilzomib single-drug treatment, and combination treatment for 48 h in MDA-MB-231. **G** Cell viability of DMSO, vorinostat, carfilzomib single-drug treatment, and combination treatment for 48 h in MDA-MB-231. **H** Cell viability of DMSO, chloroquine, and VPA single-drug treatment, and combination treatment for 48 h across OvCa cell lines (PT340, PT412, A2780, each point represents average response for each line). **I** Proteasome activity in control and 48-h vorinostat-treated OvCa cell lines (PT340, PT412, and A2780). To confirm specificity, proteasome activity was suppressed by the proteasome inhibitor MG132. **J** Proteasome activity in primary quiescent, and proliferating (vital dye bright and dim, respectively) PT340 cells. **K** Western blotting probed ATG5 in PT340 cells treated with DMSO, serum starvation, and vorinostat. Cell number/viability and proteasome studies were completed in triplicate, with *p* values calculated using two-tailed Student’s *t* tests, comparing means between independent studies.

Many of the altered pathways identified in our RNA-Seq studies, including ribosome biogenesis, RNA processing, translation initiation, and the UPS, regulate cellular proteostasis [43]. To study whether proteostasis is essential for quiescent OvCa cells’ survival, we screened five therapeutics that disrupt proteostasis: carfilzomib (a proteasome inhibitor), tanespimycin (an HSP90 inhibitor), hydroxychloroquine (an autophagy inhibitor), pidnarulex (an rRNA synthesis inhibitor), and seclidemstat (an LSD-1 inhibitor) (Table 1). We tested each drug, alone and in combination with vorinostat. Treatment with tanespimycin, carfilzomib, hydroxychloroquine, and seclidemstat as single agents led to a 20–30% reduction in cell numbers, while a ~75% reduction in cell numbers was evident after pidnarulex administration; none impacted cell viability (Fig. 6A, B, Supplementary Fig. S6A, B). In contrast, when combined with vorinostat to induce quiescence, carfilzomib, hydroxychloroquine, and pidnarulex significantly reduced cell viability (Fig. 6B, Supplementary Fig. S6B).

**Table 1:** HDACi and proteostasis targeting drugs and doses.

Compound	Description	Dose
Vorinostat	HDAC inhibitor	3 µM
Mocetinostat	HDAC inhibitor	1 µM
Belinostat	HDAC inhibitor	1 µM
VPA	HDAC inhibitor	3 mM
Hydroxychloroquine	Increase pH within intracellular vacuoles, affect the function of lysosome, endosomes, and Golgi apparatus to inhibit autophagy [81]	50 µM in PT340 30 µm in PT412 and Hey1
Chloroquine	Autophagy inhibitor as above	100 µM
Tanespimycin	HSP90 inhibitor [79]	30 nM
Carfilzomib	Proteasome inhibitor [80]	20 nM in PT340 and PT412 10 nM in Hey1 and MDA-MB-231
Pidnarulex	rRNA synthesis inhibitor [82]	150 nM
Seclidemstat	Lysine-specific demethylase 1 (LSD1) inhibitor [83]	300 nM

The vorinostat-carfilzomib combination resulted in the most significant cell death. This result was striking as neither drug alone was cytotoxic. Indeed, a synergy calculation using Combenefit [44] with the HSA model demonstrated that vorinostat and carfilzomib had profound synergistic effects (synergy index 25–73) on OvCa cell viability (Fig. 6C). Confirming the importance of HDAC inhibition on the effect of combination therapy, mocetinostat (Fig. 6D, E, Supplementary Fig. S6C) and belinostat (Supplementary Fig. S6D, E) similarly showed synergistic effects with carfilzomib in OvCa cell lines. To determine whether this effect was ovarian cancer specific, we repeated these studies with the MDA-MB-231 breast cancer cell line. Similarly to the outcome in OvCa lines, HDAC inhibitor treatment also decreased MDA-MB-231 cell growth while having minimal impact on cell viability, and combination therapy with vorinostat and carfilzomib significantly increased cell death (Fig. 6F, G).

To further confirm a critical role for proteostasis in the survival of HDACi-induced quiescent cells, we performed similar combinatorial studies of HDACi with the autophagy inhibitor chloroquine. For these studies we chose to use the HDACi valproic acid as it is more economically viable for patient use and, thus, when combined with chloroquine, could provide an easier path to the clinic. While neither agent demonstrated significant single-agent toxicity, the chloroquine and VPA combination resulted in a 50% reduction in cell viability across three OvCa cell lines (PT340, PT412, and A2780; Fig. 6H).

To determine why vorinostat-induced quiescent cells are sensitive to proteasome inhibitors, we measured the proteasome activity in control and vorinostat-treated cells by monitoring the liberation of 7-amino-4-methylcoumarin from the synthetic proteasome substrate Suc-LLVY-AMC [45]. The data shown in Fig. 6I indicates that vorinostat-treated OvCa cells had increased proteasome activity compared to the DMSO control. To show that this relationship mirrored proteasome activity in primary quiescent cells, we similarly evaluated proteasome activity in proliferating and quiescent primary cells, based on CTV vital dye stain. CTV-bright cells/non-proliferating also demonstrated an increase in proteasome activity proportional to the increase in activity in vorinostat-treated cells (Fig. 6J). Similarly, analysis of the autophagy marker ATG5 revealed potential autophagy upregulation in both serum-starved and vorinostat-induced quiescent cells (Fig. 6K). Combined, our data suggests that proteasome activity and autophagy are upregulated in quiescent OvCa cells, which may be essential to support cell viability.

### HDACi and a proteostasis inhibitor act synergistically in therapeutic tumor models

**Fig. 7:**
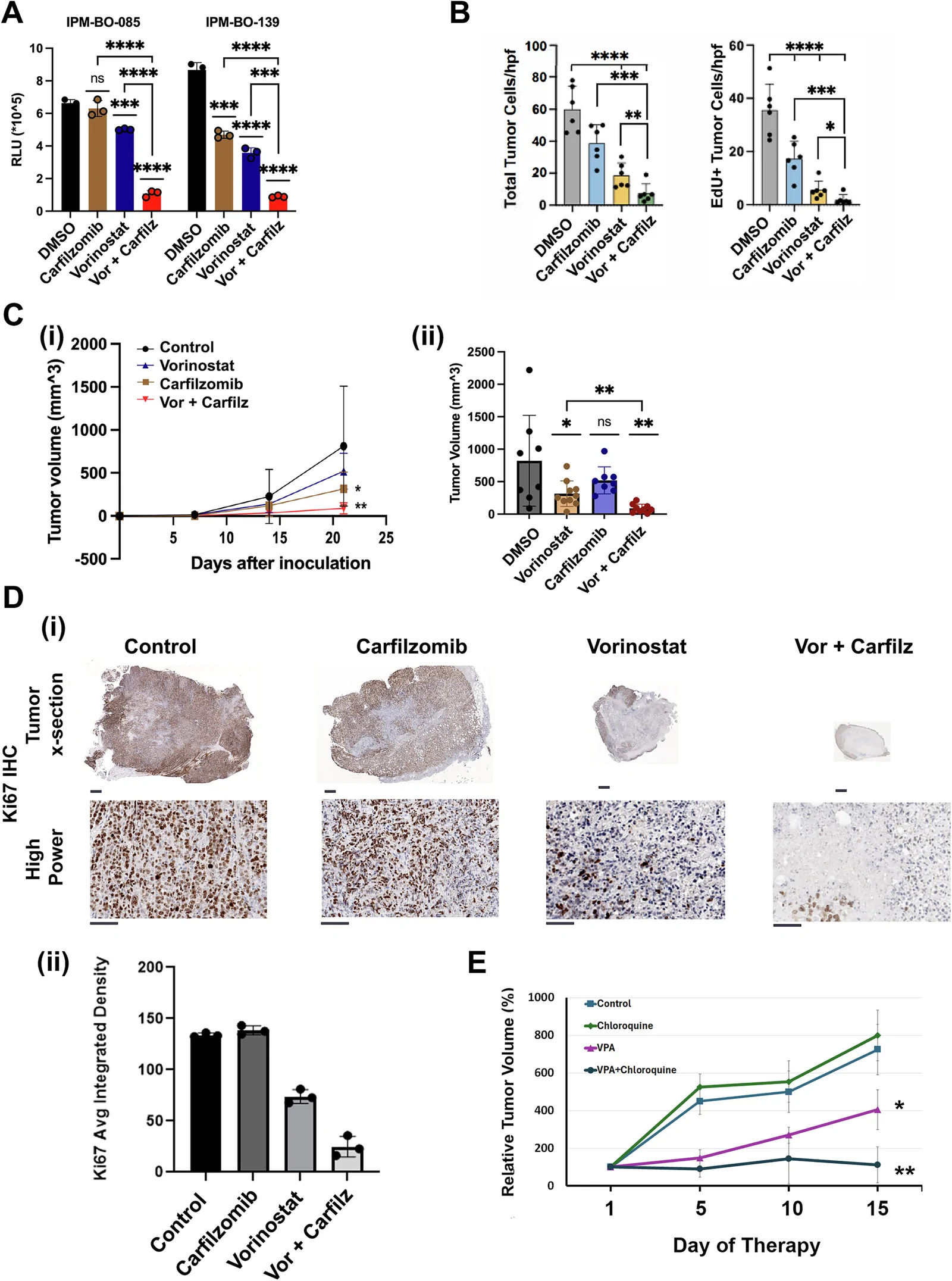
Combined targeting of proteasome and NuRD complex delays tumor growth in vivo. **A** Cell viability, as assessed by CellTiter-Glo 3D, of patient-derived triple-negative breast cancer organoids treated with DMSO, vorinostat, carfilzomib single-drug treatment, and combination treatment. **B** Total and EdU+ viable MDA-MB-231 breast cancer cells co-cultured with hepatocytes and treated with the indicated therapies. Data are pooled results from replicate experiments compared with Student’s *t* test. **C** PT340 cells were injected subcutaneously and bilaterally into the flanks of 5 mice and grew for 5 days before drug treatment. Tumor growth curves and final tumor volumes from NSG mice injected with PT340 cells subcutaneously (DMSO *n* = 8, SAHA *n* = 10, Carfilzomib *n* = 8, SAHA + Carfilzomib *n* = 9, non-subcutaneous tumors were excluded). Mice were treated with vehicle control, vorinostat, carfilzomib single-drug treatment, and combination drugs 5 days after inoculation. **D** (i) Low-magnification (whole tumor cross-section) and high-power images of Ki67 staining indicate treatment groups, and (ii) quantification of Ki67 average integrated density from 10 high-power sections of three tumors per treatment group. **E** Tumor volume curve of NSG mice injected with PT340 cells subcutaneously (*n* = 10 tumors). Mice were treated with vehicle control, valproic acid (VPA) or chloroquine single-drug treatment, and combination VPA and chloroquine 5 days after inoculation.

We next evaluated the therapeutic effect of single-agent and combination therapy ex vivo and in vivo. First, two patient-derived triple-negative breast cancer organoid models (IPM-BO-085 and IPM-BO-139) were treated with DMSO, vorinostat, or carfilzomib, or a combination of the two drugs. The viable cell number was then assessed with the CellTiter-Glo 3D cell viability assay. Single-agent vorinostat had no effect in IPM-BO-085 organoids but reduced growth by ~50% in IPM-BO-139 organoids. In contrast, single-agent carfilzomib reduced growth by 20% and 60%, respectively, in the two models. However, a combination of vorinostat and carfilzomib treatment significantly reduced organoid viability in both models, with an 85–90% reduction in viable cell number (Fig. 7A).

Next, we evaluated the therapeutic response in an ex vivo 2D metastasis model, using a co-culture of primary murine hepatocytes and RFP-labeled MDA-MB-231 cells. Cells were co-cultured for 24 h and then treated with DMSO, vorinostat (1.5 μM), carfilzomib (10 nM), or the combination for 72 h before the total number of viable cancer cells and EdU+ cancer cells were assessed. Single-agent vorinostat treatment was associated with a significant reduction in total and EdU+ cancer cell numbers, and mirroring the results shown above, combination therapy was again associated with significant reduction in total cell numbers and a near complete loss of EdU+ cells (Fig. 7B).

We then evaluated single and combination drug therapy in vivo, using a subcutaneous ovarian PDX model. PT340 cells were injected into the flanks of NSG mice, and daily drug treatments were started 5 days after inoculation. Single-agent carfilzomib did not significantly impact tumor growth, but consistent with an induction of quiescence, single-agent vorinostat significantly slowed tumor growth, while—consistent with tumor cell killing—combination therapy with vorinostat and carfilzomib was highly effective at limiting tumor growth, with tumors undetectable in two of nine mice (Fig. 7C). Immunohistological analysis of tumors for Ki67 expression also demonstrated that >90% of cells in the control and carfilzomib treatment groups were proliferative, with less than 50% proliferative in the reviewable vorinostat-treated tumors. Dual therapy-treated tumors showed a profound reduction in Ki67 stand-in and large areas of necrosis (Fig. 7D).

To confirm the therapeutic benefits of combination quiescence-inducing/proteostasis-targeting drugs, we repeated the in vivo study with valproic acid and chloroquine. Like the results shown above, single-agent chloroquine (proteostasis-targeting agent) had no treatment effect, while single-agent valproic acid (quiescence-inducing agent) significantly delayed tumor growth. However, combination therapy again prevented tumor growth and, in fact, induced mild tumor regression (Fig. 7E).

## Discussion

We report here that the downregulation or inhibition of NuRD complex components contribute to induction of quiescence in OvCa by modulating the RHO GTPase pathways and driving cytoplasmic localization of MRTFA. We also report that quiescent OvCa cells exhibit dependencies related to altered proteostasis, including increased proteasome activity. Importantly, the increase in proteasome activity appears to be an Achilles’ heel for quiescent cells. While bulk OvCa cells are, in general, therapeutically resistant to proteasome inhibition, HDACi-induced quiescent cells are killed when a clinical proteasome inhibitor is added. Finally, by overlaying RNA-Seq data sets from quiescent cancer cells and yeast, we propose the existence of a core quiescent cell signature. This signature was validated in quiescent cells across several cancer types.

### The NuRD complex and quiescence

We found that MBD3 and CHD4, two components of the NuRD complex, are downregulated in primary quiescent cells at both the RNA and protein level. Inhibition of the activity of NuRD complex via either genetic knockdown or pharmacologic inhibition with HDACi can enforce a quiescent state in OvCa cells. However, the effect of knockdown was milder than that of pharmacologic inhibition. We speculate this is due to the redundancy of CHD/MBD family proteins. CHD3, CHD4, and CHD5 are three highly homologous proteins and can form distinct NuRD complexes that contain other components [46–48]. Since CHD5 is preferentially expressed in testis and the nervous system, CHD3 and CHD4 are more commonly present in the NuRD complex [48, 49]. Studies revealed that CHD3-NuRD and CHD4-NuRD have different localization patterns and distinct regulated genes, but there are common target genes equally influenced by CHD3-NuRD and CHD4-NuRD [46]. Likewise, MBD2 and MBD3 have been reported to form mutually exclusive complexes with different functions but can also bind to the same promotor regions [50, 51]. Furthermore, evidence showed that, in the absence of CHD4, other components can form a stable complex with robust HDAC activity, indicating that CHD4 is a peripheral component in the NuRD complex [52]. Therefore, knockdown of a single CHD/MBD is insufficient to fully inhibit NuRD complex activity.

Administration of several HDACi was found to strongly induce quiescence in OvCa cells, and indeed, they are proven to have anti-tumor effects in multiple other cancers [53–55]. Vorinostat, which inhibits the activity of class I and II HDACs, was the first FDA-approved HDACi used for refractory cutaneous T-cell lymphoma [55, 56]. Consistent with our results, vorinostat treatment induced cell cycle arrest in breast cancer and in non-small cell lung cancer cell lines [57, 58]. Others have reported an effect in OvCa cells; however, the phase of cell cycle arrest is controversial [59–61]. We found that HDACi treatment was associated with downregulation/change of phosphorylation of numerous critical cell cycle regulators (CCNA2, E2F6, pRB, pS6K, p27).

Previous studies have shown that HDAC inhibitors affected the cell cycle through multiple mechanisms. By increasing histone acetylation and altering the transcription of key regulators, HDACi regulate expression of cell cycle regulators such as p21 and p27 [62, 63]. HDACi also modified the acetylation of p53 [64], thus affecting the p53-p21-Rb pathway. Moreover, with genotoxins and microtubule poisons, it can trigger a G2 checkpoint by DNA-damage–responsive [65]. We found that HDACi was associated with closed chromatin and several genes associated with the RHO/RAC GTPase cycle. A RAC1 specific inhibitor also induced quiescence, suggesting that the RAC1 GTPase cycle contributes to the quiescent phenotype. Furthermore, both HDACi and RAC1i treatment resulted in the loss of nuclear localization of MRTFA, a transcription factor known to cooperate with SRF to drive cellular proliferation [39]. Interestingly, we recently reported that MRTFA inhibition drives cellular quiescence [22]. This suggests a possible feed-forward loop whereby HDAC inhibitions in MRTFA inhibition, which further downregulates NuRD components to amplify the quiescence response. Furthermore, as MRTFA-SRF pathway inhibition has been linked with metastasis [66], RACi could similarly impact metastasis. Additional studies will be necessary to validate this hypothesis.

Although HDACi, such as vorinostat, have demonstrated activity in pre-clinical solid tumor models, as single agents in phase II clinical trials for solid tumors—including breast cancer, colorectal cancer, non-small cell lung cancer, or epithelial ovarian cancer—they have not led to tumor regression [67–69]. HDACi induction of quiescence would be consistent with these results; induction of quiescence would be expected to slow tumor growth or, at best, induce stable disease, but would be unable to reduce tumor volume. As most clinical trials have primary endpoints related to radiologic reduction in tumor volume, these trials were destined to fail. Our results instead suggest HDACi could be used as a maintenance therapy to delay recurrence. Some studies have combined HDACi with chemotherapy. A combination of belinostat, carboplatin, and taxol demonstrated a surprisingly high (54%) response rate in platinum-resistant ovarian cancer [70]. Timing of the administration of an HDCAi and chemotherapy will be critical. Given that quiescent cells are typically chemotherapy resistant, our results further suggest that HDACi should not be given prior to chemotherapy. However, HDACi after chemotherapy could be used to prevent cancer cell proliferative recovery either between cycles of chemotherapy or could be used as maintenance after chemotherapy. Furthermore, recent studies suggest that HDAC1 and HDAC2 play a potential role in epigenetically priming chemoresistant ‘persister’ cells [71]. Thus, targeting HDCAi, used in such a manner, could reduce this persister cell population, which is thought to drive disease recurrence.

### Quiescence and proteostasis

Our studies are consistent with both primary quiescent cells and vorinostat-driven quiescent cells exhibiting enhanced proteasome and autophagy activity. Our results are also consistent with other work indicating that vorinostat induces proteasome activity [72–76] and are in line with prior studies noting that proteasome-related pathways are altered in quiescent cells [23]. The upregulation of these proteostasis pathways indicates they may support quiescence. Indeed, the preclinical studies performed here demonstrate that proteasome activity supports quiescent cancer cell survival. As such, approaches targeting these pathways could help eradicate residual chemotherapy-resistant, quiescent cancer cells and could have a profound impact on cancer patient outcomes. The fact that similar treatment effects were also noted in breast cancer cells suggests that this is not a cancer type-specific effect. It is interesting to note that proteasome inhibitor therapy has proven most effective in multiple myeloma – a “smoldering,” more quiescent cancer type [77]. Similarly, given that neurons are generally considered quiescent cells, it may not be a coincidence that neurotoxicity is the major side effect of proteasome inhibitors.

Previous studies have shown that proteasome inhibitors can act synergistically with HDAC inhibitors in multiple myeloma. In a study of patients with relapsed or relapsed and refractory multiple myeloma, adding the pan-HDAC inhibitor panobinostat to bortezomib and dexamethasone significantly improves progression-free survival [78]. Another study in patients with multiple myeloma showed higher response rates and prolonged progression-free survival (PFS) with vorinostat and bortezomib combination compared with bortezomib alone [79]. A phase I study of the combination of carfilzomib and panobinostat in relapsed/refractory multiple myeloma suggested safety of the combination and resulted in a PFS of 8 months and overall survival of 23 months, supporting the clinical feasibility of dual proteasome–HDAC inhibition [80]. Moreover, several clinical trials are evaluating combinations of proteasome and HDAC inhibitors in solid tumors. A phase I study of vorinostat plus the proteasome inhibitor marizomib in Phase 1 clinical trial patients with melanoma, pancreatic carcinoma, or Non-small Cell Lung Cancer achieved stable disease in 61% and decreased tumor size in 39% of evaluable patients [81]. Likewise, a phase I trial of vorinostat combined with bortezomib in advanced solid tumors reported that five patients had evidence of stable disease after the cycle two disease evaluation among 29 patients [82]. This therapeutic strategy has not yet been investigated in women with gynecologic malignancies. Given our data and the encouraging data for belinostat combined with carboplatin and taxol [70], we are currently exploring a clinical trial approach combining HDACi and proteostasis inhibition as a window of opportunity study in ovarian cancer.

Another proteostasis-related pathway, autophagy, has been shown to be dysregulated in cancer stem cells [83]. We found that a key autophagy-associated protein is upregulated in qOvCa cells, and dual HDACi therapy and autophagy inhibition with hydroxychloroquine or chloroquine induced OvCa cell death in vitro and delayed tumor growth in vivo. Parallel to our findings, Rehman et al. revealed that a reversible drug-tolerant persister state, induced by CPT-11, is maintained by an upregulated autophagy pathway in colorectal cancer cells [84]. Specifically, they demonstrated that a combination of chloroquine and CPT-11 robustly increased apoptosis.

### Quiescent cell core signature

Our results and others indicate that quiescent cells may have a unique biology that can be exploited for therapeutic benefit. Therefore, to identify additional targets in quiescent cancer cells, we identified genes in common between quiescent cancer cells and quiescent yeast. This core quiescent signature suggests several new therapeutic targets, including several solute carriers and cell cycle-related genes. In the yeast studies, some genes were identified in response to quiescence-driving stimuli, while others were necessary for “mitotic competency” or the ability to replicate after quiescence [41, 42]. We speculate that these mitotic competency genes may include genes that help quiescent cells re-enter the cell cycle. Further studies will be needed to confirm this hypothesis.

### Limitations and future directions

Our study has several limitations that should be considered when interpreting these findings. A key limitation of our study is that most experiments rely on pharmacologic HDAC inhibition, which has broad, genome-wide effects on chromatin and transcription and therefore does not provide specificity for the NuRD complex. Although we observe downregulation of NuRD components and correlate this with HDACi-induced quiescence and MRTFA/SRF pathway modulation, without rescue experiments, these data do not establish NuRD as a unique or critical mediator of the phenotype. Second, our in vivo experiments were performed in a single PDX model with a relatively short treatment and follow-up period. A longer treatment and follow-up observation in vivo could provide a better insight into treatment efficacy. Third, the core quiescence signature is derived from transcriptomic overlap across species and has not been functionally verified. The loss- and gain-of function analysis of these quiescence core genes are planned for the future.

In summary, this study provides insight on the NuRD complex’s potential function in regulating cancer cell quiescence. By perturbing NuRD complex activity, OvCa cells showed a decreased proliferation rate but with high cell viability and the ability to regrow rapidly after drug removal. Increased proteasome activity helps qOvCa cells maintain proteostasis and survive under stress. With combinatorial inhibition of HDACs and proteasome activity, most OvCa cells were killed in vitro. The combination therapy of vorinostat and carfilzomib in vivo showed profound effects in delaying tumor growth, which indicates a possible new therapeutic method for OvCa patients.

### Ethics approval and consent to participate

No primary human samples were used in this work. The experimental techniques were conducted with the criteria established by the Institute for Laboratory Animal Research of the National Academy of Sciences and were approved by the University of Pittsburgh IACUC Protocol #: 23114036. All experimental approaches were approved by the University of Pittsburgh Institutional Biosafety Committee (IBC) protocol #IBC201700278.

## Materials and methods

### Cell lines and culture

PT340 cells were derived from an abdominal metastasis of a mixed high-grade serous/clear cell ovarian cancer [27]. PT412 was a patient-derived, stage III, abdominal metastasis, high-grade serous ovarian cancer cell line from Dr. Geeta Mehta [85]. Hey1, H522, A2780, and MDA-MB-231 were obtained from ATCC. All cell lines were cultured in RPMI-1640 media, supplemented with 10% FBS and 1% Pen Strep, in the cell incubator at 37 °C, with 5% CO_2_.

### Constructs and transfection

Scramble, MBD3, and CHD4 siRNAs were obtained from Qiagen (1027280, SI00024556, SI00024563, SI00057862, SI04439848). Lipofectamine 3000 transfection reagent (Invitrogen Cat. L3000001) was used to transfect siRNAs in OvCa cells. For each well in a 6-well plate, 5 µl of lipofectamine 3000 were used to transfect 60 nM indicated siRNA. Media was changed 6 h after transfection, and cells were collected at 72 h after transfection for analysis. Scramble, MBD3, and CHD4 shRNAs were purchased from Sigma (T SHC016, RCN0000016153, TRCN0000016154, TRCN0000021361, TRCN0000021363). All shRNAs were stably transduced in OvCa cells by lentivirus.

### Quantitative polymerase chain reaction

RNeasy Mini Kit (Qiagen Cat. 74104) was used to extract RNA, and SuperScript III First-Strand Synthesis System (Invitrogen Cat. 18080051) was used to generate cDNA. qPCR was performed using SsoAdvanced Universal SYBR Green Supermix (BIO-RAD Cat. 1725270). Primers used are listed in Table 2.

**Table 2:** Primers sequences.

Gene name	Forward primer	Reverse sequence
CHD4	TGAGGGCAGCGACTATACTCC	GAGCAGATGATTTAGGCTCCTTT
MBD3	CTGAGCACCTTCGACTTCCG	CCGGCTGCTTGAAGATGGA
CCNA2 CLSPN ORC1 E2F8 MYBL2 CD24 APOE UBE2L6 FBOX2 PSMB9	GGATGGTAGTTTTGAGTCACCAC TGGAGAGTGGGGTCCATTCAT TGGATCGAAAACTGCACTACC CCTGAGATCCGCAACAGAGAT CTTGAGCGAGTCCAAAGACTG CTCCTACCCACGCAGATTTATTC GTTGCTGGTCACATTCCTGG CTGGAAGCCTTGCACCAAGA GTGTCGCAAAGCACAGGTC GCACCAACCGGGGACTTA	CACGAGGATAGCTCTCATACTGT CCGGGGTTTACGTTTGAAGAAA CTGGATGTGAATCTCGGTGGA AGATGTCATTATTCACAGCAGGG AGTTGGTCAGAAGACTTCCCT AGAGTGAGACCACGAAGAGAC GCAGGTAATCCCAAAAGCGAC GAACATGAGTTAGGAGGGCCG CGGACAGTAGCTTAACGGTGAG CACTCGGGAATCAGAACCCAT

### Western blotting and antibodies

For validation of downregulation of MBD3 and CHD4 in serum starvation-induced quiescent cells, cells were treated with normal media and serum-withdrawal media for 72 h. For testing shRNA efficiency, cells were seeded and cultured for 72 h. Then, they were treated with trypsin and collected. Cells were lysed with RIPA buffer (Thermo Scientific Cat. 89901) supplemented with Halt Protease and Phosphatase Inhibitor Cocktail (Thermo Scientific Cat. 1861281) and followed by sonication. Protein samples were mixed with Laemmli Sample Buffer (BIO-RAD Cat. 1610747) before heating at 95 °C for 7 min. Samples were run on 4–12% NuPAGE Bis-Tris Gel (Invitrogen) in NuPAGE MOPS SDS Running Buffer (Invitrogen Cat. NP0001-02) at 120 V for 90 min and transferred to a 0.45 µm polyvinylidene difluoride membrane in NuPAGE Transfer Buffer (Invitrogen Cat. NP00061) at 30 V for 90 min. SeeBlue Plus2 Pre-Stained Protein Standard (Invitrogen Cat. LC5925) was used as marker. Membranes were blocked in 5% milk in TBST buffer for 1 h at room temperature and incubated with 1:1000 anti-MBD3 (Abcam, Ab157464), 1:1000 anti-CHD4 (Proteintech, 14173-1-AP), 1:1000 anti-Histone H3 (Proteintech, 17168-1-AP), 1:1000 anti-LC3B (Proteintech, 18725-1-AP), 1:1000 ATG5 (Cell Signaling, 2630), 1:1000 Ku70 (Cell Signaling, 4588) in 5% milk overnight at 4 °C. After washing with TBST three times (10 min each), membranes were incubated with 1:10,000 anti-rabbit HRP (Cell Signaling, 7074S) for 1 h at room temperature. After washing with TBST three times (10 min each), Pierce ECL Western Blotting Substrate (Thermo, 32106) and SuperSignal West Femto Maximum Sensitivity Substarte (Thermo, 34095) were used.

### Pharmacological treatments

Vorinostat (S1047), mocetinostat (S1122), belinostat (S1085), carfilzomib (S2853), tanespimycin (S1141), hydroxychloroquine, pidnarulex (S2684), and seclidemstat (S6722) were purchased form Selleckchem. Chloroquine diphosphate salt (C6628-25G) and valproic acid sodium salt (P4543-10G) were purchased from Sigma-Aldrich. Each drug was dose-titrated, and a dose that did not induce cell death was chosen (Table 1). NSC 2376, Rhosin hydrochloride, and ML-141 were purchased from Tocris Bioscience and used for daily treatment at the indicated doses.

### Cell proliferation-cell counts

Cell counts were performed by using the Brightfield app on a CellDrop automated cell counter (Denovix).

### Apoptosis assay and flow cytometry

Cells were cultured with indicated conditions for 48 or 72 h. Floating cells in cultured media and attached cells were collected and mixed. Using BD Pharmingen FITC Annexin V Apoptosis Detection Kit I (BD Biosciences, 556547), cells were stained with Annexin V-FITC and propidium iodide (PI)-PE. CytoFLEX flow cytometer (Beckman-Coulter) and FlowJo were used to analyze samples (20,000 events per sample).

### CellTrace violet staining

PT412 were labeled with CellTrace Violet (Thermo, C34557) as per the manufacturer’s protocol and cultured for 7 days. Flow cytometry was then used to identify CTV-bright and CTV-dim cells.

### Fucci system

Hey1-Fucci cells expressed p27K-mVenus and CDT1(30/120)-mcherry [27]. Cells in G0 state express both p27K-mVenus and CDT1(30/120). After DMSO, 3 µM vorinostat, 1 µM belinostat, 1 µM mocetinostat, and 3 mM VPA treatment for 48 h, flow cytometry was performed.

### Multiplexed immunofluorescence and cell cycle mapping

PT340 were seeded in the 8-well chamber (ibidi, 80841) and treated, with or without 3 µM vorinostat, for 48 h. After treatment, cells were fixed with paraformaldehyde for 15 min at room temperature, followed by permeabilization with 0.1% Triton X-100 in PBS for 10 min and blocking in 10% (w/v) donkey serum and 3% (w/v) bovine serum albumin (BSA) in PBS for 1 h at room temperature. Primary antibodies were diluted in blocking solution and incubated for 1 h at room temperature (anti-c-Myc, Abcam, ab190560; anti-p16, Abcam, ab192054; anti-cyclin E2, Abcam, ab207336; anti-phospho-H2AX, Abcam, ab206900; anti-beta-catenin, Millipore, C7738; anti-CDC6, Abcam, ab211734; anti-CDC25C, Abcam, ab205425; anti-CDK2, Bio-Techne, AF4654; anti-CDK4, Abcam, ab213216; anti-CDK6, Abcam, ab198946; anti-cyclin A2, Abcam, ab217731; anti-cyclin B1, Abcam, ab214381; anti-cyclin B2, Abcam, ab250841; anti-cyclin D1, Abcam, ab203448; anti-cyclin D3, Abcam, ab245734; anti-cyclin E1, Abcam, ab194069; anti-E2F1, BioLegend, BL606052; anti-p27, Abcam, ab206927; anti-p53, Abcam, ab224942; anti-phospho-AKT, Cell Signaling Technology (CST), 4075; anti-PCNA, CST, 82968; anti-phospho-ERK, CST, 45899; anti-Plk1, Abcam, ab223901; anti-phospho-RB, CST, 8974; anti-phospho-S6, CST, 3985; anti-phospho-STAT3, CST, 4324). In-house primary antibody fluorescent conjugates were generated using the Alexa Fluor 647 labeling kit (Invitrogen, A20186) or the Alexa Fluor 555 labeling kit (Invitrogen, A20187). Nuclei were labeled with Hoechst (2 µg/mL, Invitrogen, H3570) for 10 min at room temperature and imaged in 50% glycerol in PBS. Following imaging, fluorescent dyes were inactivated using an alkaline solution containing H₂O₂ for 15 min with agitation, followed by a PBS wash. Samples were re-imaged to measure residual fluorescence. Subsequent rounds of antibody staining and imaging were then conducted as previously described, starting with primary antibody incubation. Image acquisition, flat-field correction, autofluorescence removal, and registration were performed using the Cell DIVE Imager (Leica Biosystems). Single cells were segmented using Cellpose [28], and intensity measurements were extracted using scikit-image [29]. Feature selection was performed as previously described [30], and cell cycle maps were generated using PHATE [31] as previously described [32].

### RNA seq and ATAC Seq

PT340 and PT412 were treated with or without 3 µM vorinostat for 48 h, and RNA was extracted as described before. The following processes were performed as we described [27]. R studio (2023.12.0+369) and R packages ggplot2 (3.4.4), pheatmap (1.0.12), biomaRT (2.58.2), clusterProfiler (4.10.0), org.Hs.eg.db (3.18.0), and AnnotationDbi (1.64.1) were used for gene ontology (GO) biological process analysis and generating heatmaps and dot plots. To find yeast gene orthologs in humans, g:Orth in g:Profiler was used [86]. Venn diagram was produced by jvenn [33].

ATAC-Seq was performed in collaboration with the University of Pittsburgh sequencing core, using the OMNI-ATAC protocol, with sequencing on the NextSeq2000 instrument, with a P2/100 flowcell and 58/10/10/58 read lengths. Paired-end FASTQ files were processed with Trim Galore v0.6.10 to remove sequencing adapters, followed by alignment to hg38 with Gencode v13 annotation. Duplicates were flagged with SAMblaster v0.1.26, then removed with SAMtools v1.17, before peak calling with MACS2 v2.2.7.1 [87]. Deduped libraries averaged 60.2 million paired fragments, and deepTools v3.5.2 fingerprint plots showed an expected mapping distribution into open chromatin bins [88]. Libraries also showed a strong enrichment around transcriptional start sites (TSSs) as expected from ATAC-seq data using the TSSEscore function from the ATACseQC R package v1.32.0 [89]. Narrow peaks from MACS2 were imported into R, and a consensus peak set was generated with DiffBind v3.14.0 [90]. Next, differentially accessible chromatin regions were identified using DiffBind workflow, including normalizing for library size and adjusted *p*-value less than 0.05. Regions of differential accessibility were annotated to genes, using ChiPSeeker v1.40.0, if they were within 2 kb upstream of the TSS [91]. Enrichment testing was done using the enrichPathway function from ReactomePA v1.48.0 [92]. Data were deposited in the NCBI GEO database reviewer token GSE317616.

### Analyzing MRTFA nuclear to cytoplasmic shift

Cells were plated on cover slips overnight and then fixed and stained with anti-MRTFA (Abcam ab219981) at 1:200, DAPI. Cells were imaged for both MRTFA and DAPI, and DAPI images were used to select nuclear area and region of interest for integrated density quantification using ImageJ (https://imagej.net/ij). The MRTFA image was adjusted by threshold for the positive stain. The nuclei image was opened over the MRTFA image, and staining outside the nuclei was cleared, allowing measurement only for nuclear staining.

### Breast cancer organoid and CellTiter-Glo 3D cell viability assay

Two triple-negative breast cancer patient-derived organoid (PDO) lines were used. IPM-BO-085, which is Primary TNBC (“TNBC, neoadj AC/T, carbo, atezolizumab vs placebo”) from TP20-M196 (right breast tumor—age 45–50), and IPM-BO-139 (P10, Day 13)—Primary TNBC (“TNBC who progressed through chemo which was aborted”) from TP21-M326 (left breast tumor—age 55–60).

Cells were seeded and cultured 3 days before treatment to allow PDO reformation. Then, organoids were treated with 3 µM vorinostat, 20 nM carfilzomib, and combination for 3 days. CellTiter-Glo 3D cell viability assays (Promega, G9681) were performed as per manufacture’s protocol.

### Proteasome activity assay

Proteasome activity was measured by monitoring the synthesis of 7-amino-4-methylcoumarin from the Suc-LLVY-AMC as previously described [45], with minor modifications. In brief, PT340-Bright, and PT340-Dim cells were lysed by sonication in 50 mM ice-cold Tris-HCl pH 7.5, 1 mM DTT, 0.25 M sucrose, 5 mM MgCl2, 0.5 mM EDTA, and 2 mM ATP. The lysate was mixed with 50 µM N-SUC-LEU-LEU-VAL-TYR-7-amido-4-methylcoumarin (Sigma S6510) in 20 mM Tris-HCl pH 7.5, 1 mM ATP, 2 mM MgCl2, 0.2% BSA, in the presence or absence of 10 µM MG-132 (EMD Millipore), a proteasome inhibitor used to confirm the specificity of proteasome activity. The AMC fluorescence (RFU) was measured every 30–60 min in a Cytation 5 plate reader (BioTek) at an excitation wavelength of 340 nm and emission wavelength of 420 nm for up to 4 h.

### Breast cancer liver cell coculture

RFP-labeled MDA-MB-231 cells were seeded onto freshly isolated primary murine hepatocytes as previously described [93, 94]. After 24 h, cells were treated with DMSO, carfilzomib (10 µM), vorinostat (1.5 µM), or combination therapy. After 48 h, cells were cultured with 10 µM EdU for 24 h and then fixed with 2% paraformaldehyde at 4 °C for 30 min. EdU staining was performed using the Click-iT PLUS EdU Alexa Fluor 488 imaging kit (Life Technologies) per manufacturer’s protocol. Total number of RFP+ cells and EdU+ tumor cells was scored with three high-power fields per well [94].

### In vivo model

Female NSG mice, aged 6 weeks, were purchased and given 1 week to acclimate in the animal facility before any procedures were started. 8–10 animals per group based on historical experience that a 50% reduction in tumor growth with treatment could be detected with statistical significance. 250,000 cells were injected subcutaneously and bilaterally into the flanks in 5 mice and grew for 5 days before drug treatment. Five days after inoculation, vehicle, 90 mg/kg vorinostat, 2 mg/kg carfilzomib, combination of 90 mg/kg vorinostat and 2 mg/kg carfilzomib, 500 mg/kg VPA, 30 mg/kg chloroquine, and the combination of 500 mg/kg VPA and 30 mg/kg chloroquine were intraperitoneally injected daily for 3 weeks. Mice were not randomized as tumors were non-palpable in all mice. Mouse weight and tumor size were measured via calipers and volume calculated using the L × W × W/2 formula in a non-blinded manner. Mice were euthanized if tumor volume exceeded 2000 mm^3^ or become ulcerated. Non-subcutaneous tumors were excluded from the analysis. Tumors were formalin fixed, and paraffin embedded. Tumors were cross-sectioned and stained with anti-Ki67. Median sections were scanned and evaluated in toto across a minimum of 10 HPF for the three tumor/treatment groups, using ImageJ integrated density and means compared via two-tailed Student’s *t* tests.

### Statistical analysis and software

Statistical analysis was conducted using GraphPad Prism (10.1.1). All data were analyzed using either a using two-tailed Student’s *t* tests, or when comparing means across experimental replicates and in animal studies, an ANOVA. A minimum of three replicate experiments (*n* ≥ 3) were used for statistical analysis. “*“ represented *p* < 0.05, “**“ represented *p* < 0.01, “***“ represented *p* < 0.001, and “****“ represented *p* < 0.0001. Data were plotted with means and standard deviations. Synergy calculation was performed by Combenefit, using the HSA model [44]. Heatmaps and dot plots were generated by R.

## Supplementary information

Supplemental Figure legends

supplemental data

## Data Availability

RNA-Seq data are in the process of being submitted to the NCI GEO database and should be available at the time of final submission.
