# Supplementary material for: Targeting HDAC and proteostasis to eradicate quiescent cancer cells
Source: Cancer Gene Ther. 2026 Aug 13;33(9):1053–68. doi: 10.1038/s41417-026-01067-y (PMC13518249; doi:10.1038/s41417-026-01067-y)
Supplement: Supplementary file 1 [file 41417_2026_1067_MOESM2_ESM.pdf]

Figure S1

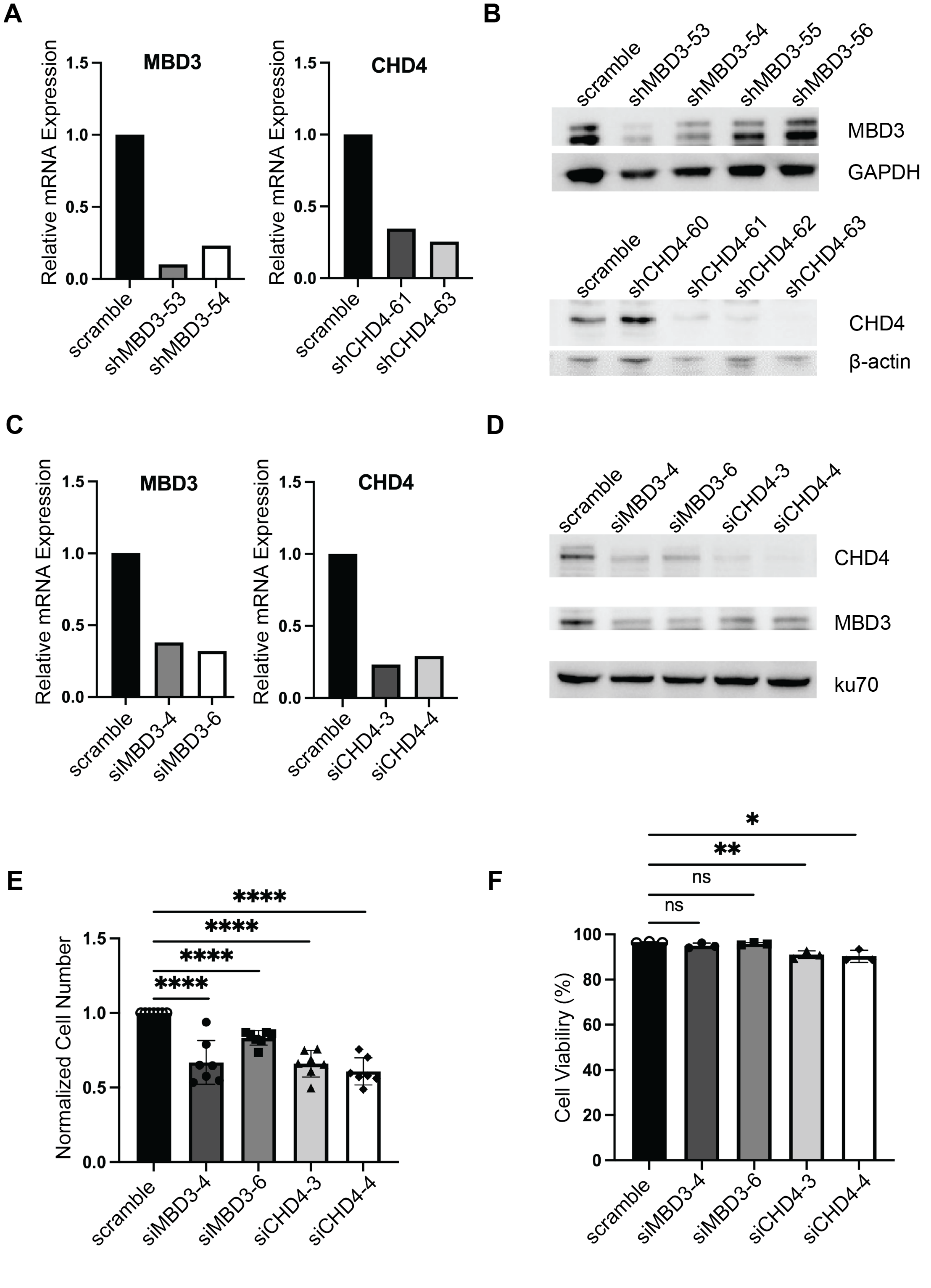

Figure S1

Figure S2

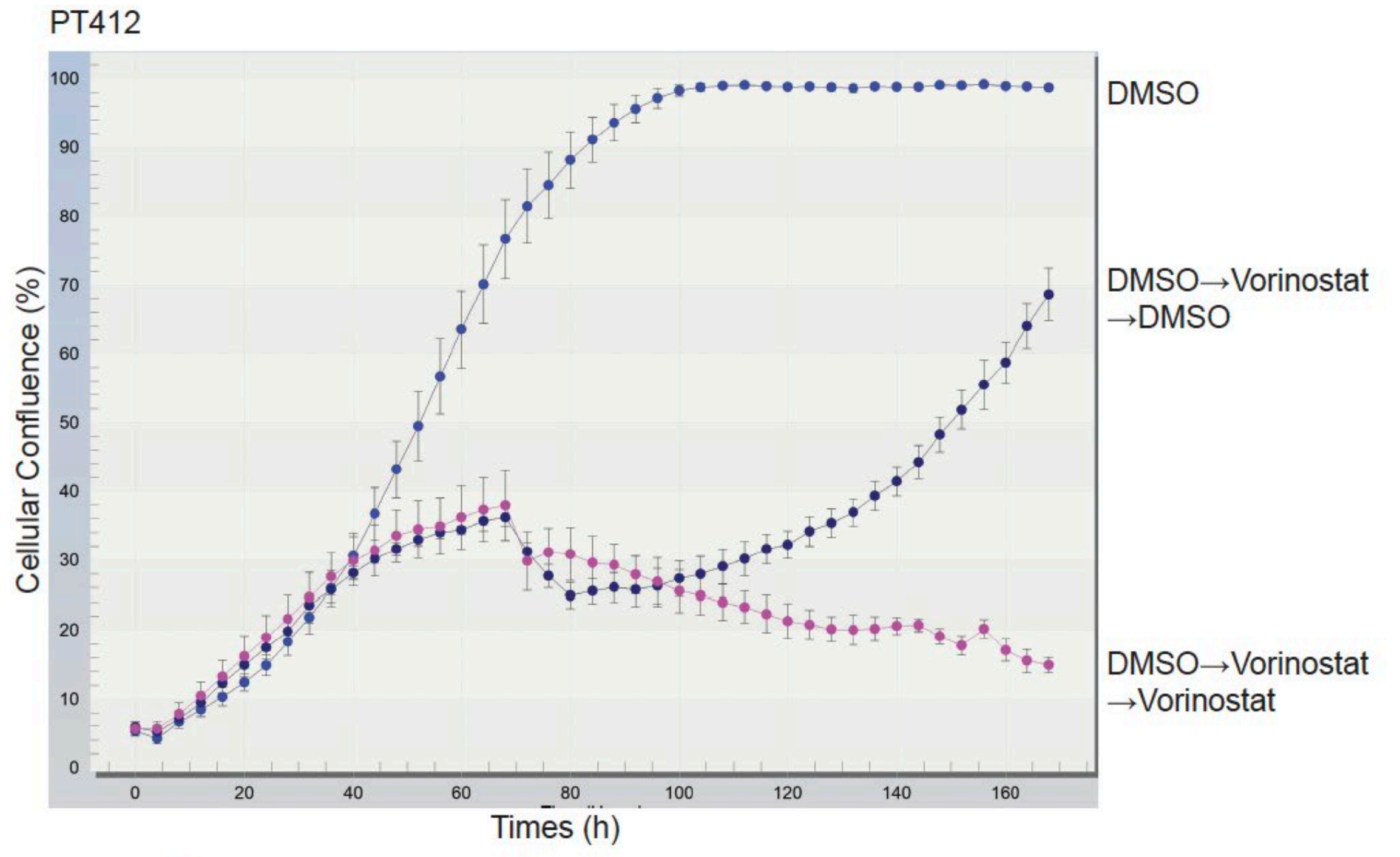

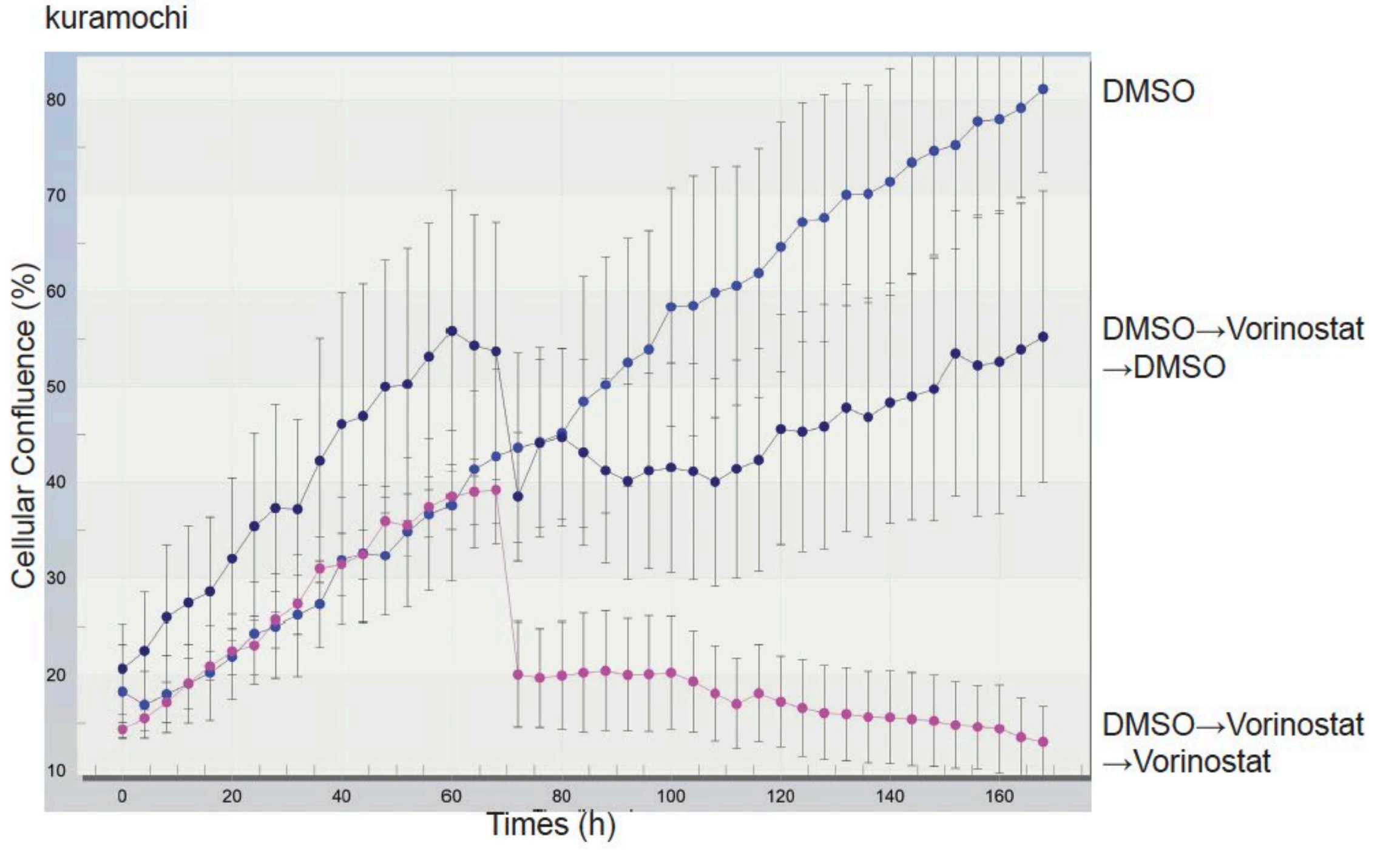

Figure S2

Figure S3

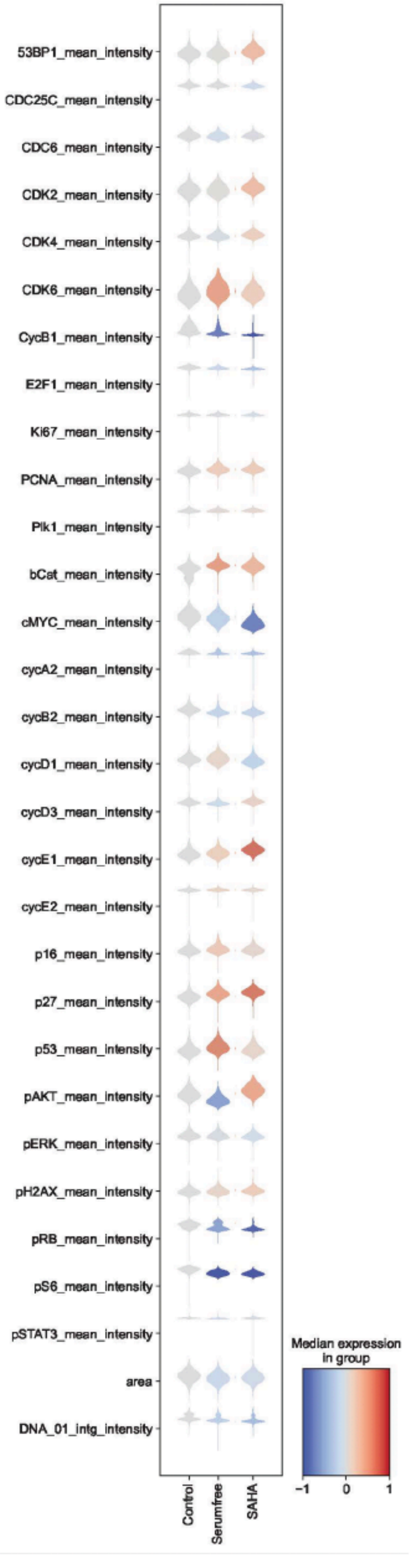

Figure S3

Figure S4

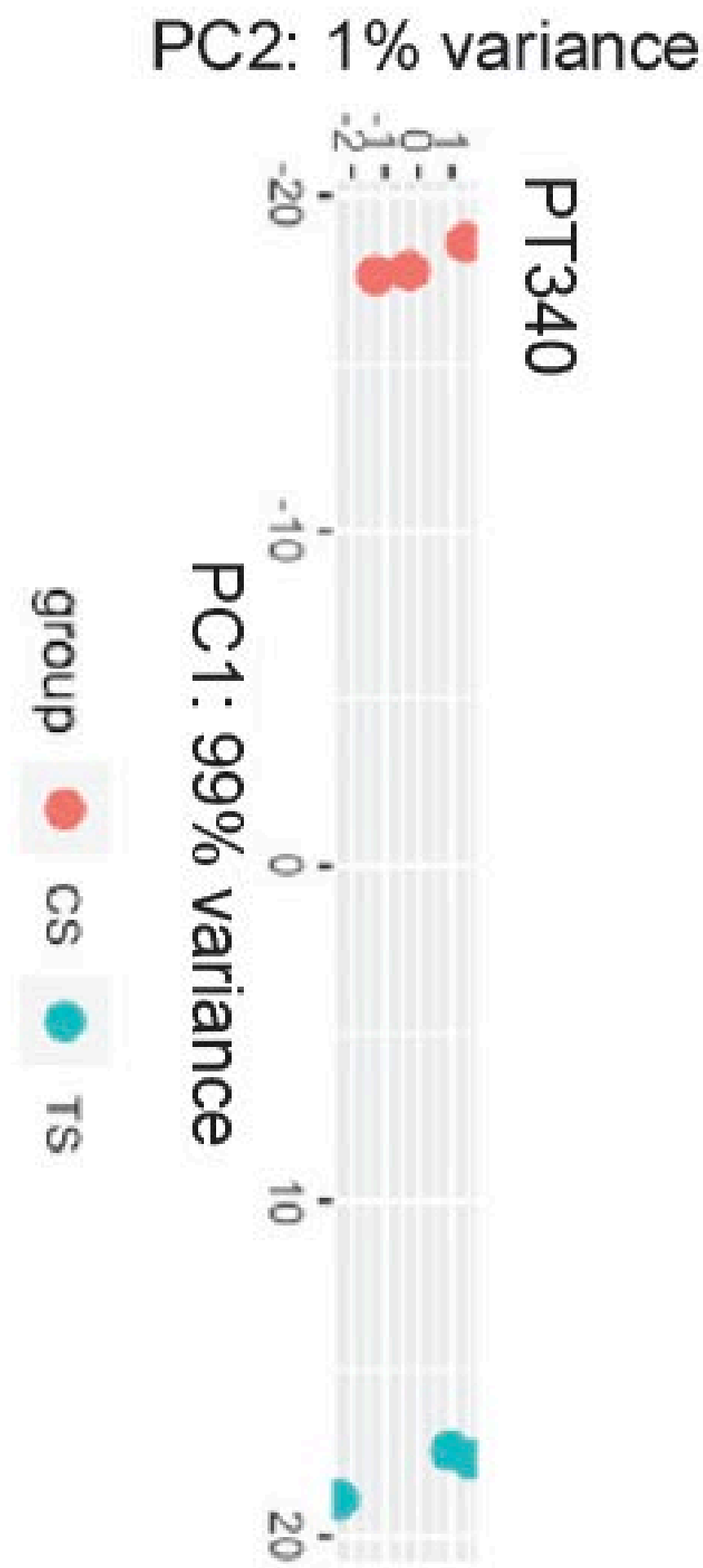
PT340
PC2: 1% variance
PC1: 99% variance
group
CS
TS
-20
-10
0
10
20
-2
-1
0
1

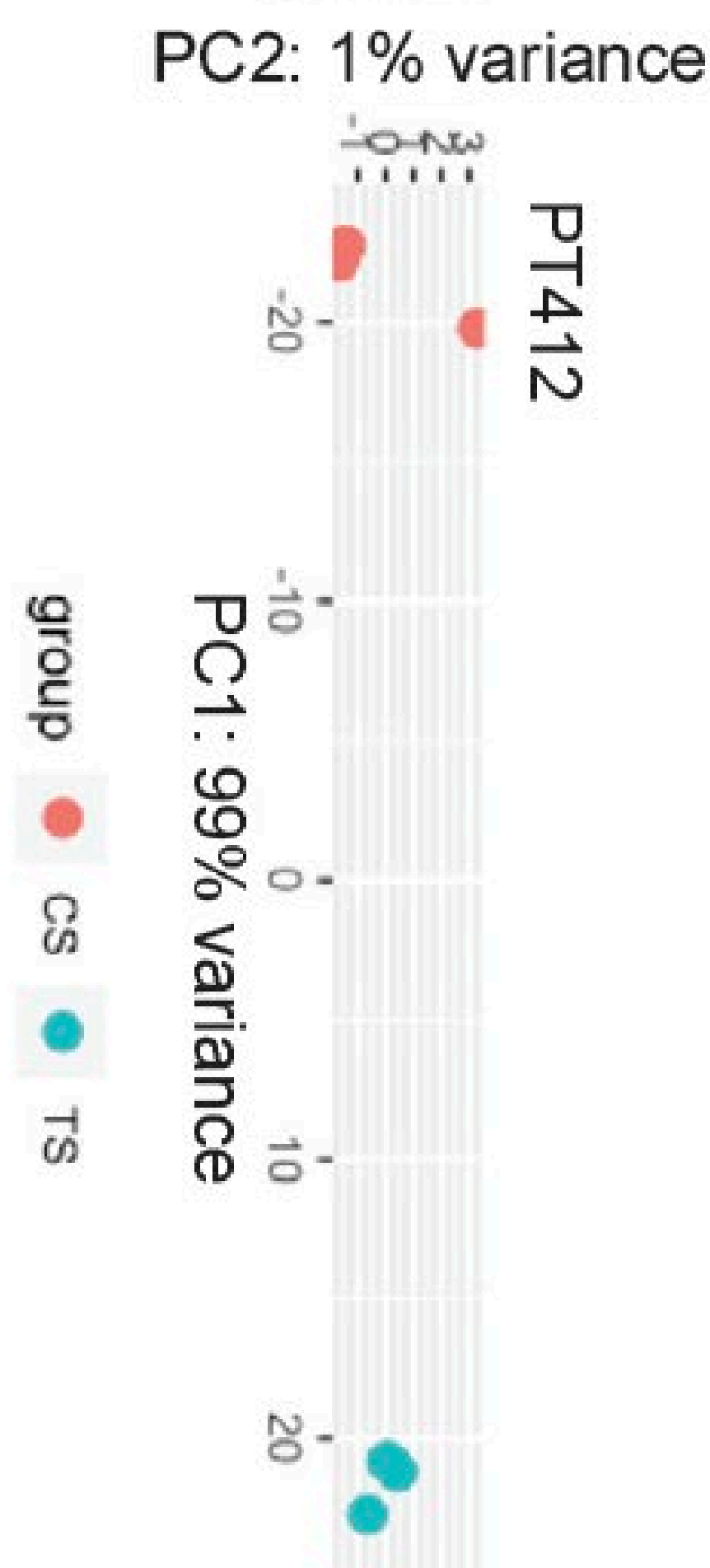
PT412
PC2: 1% variance
PC1: 99% variance
group
CS
TS
-20
-10
0
10
20
-1
0
1
2
3

Figure S5

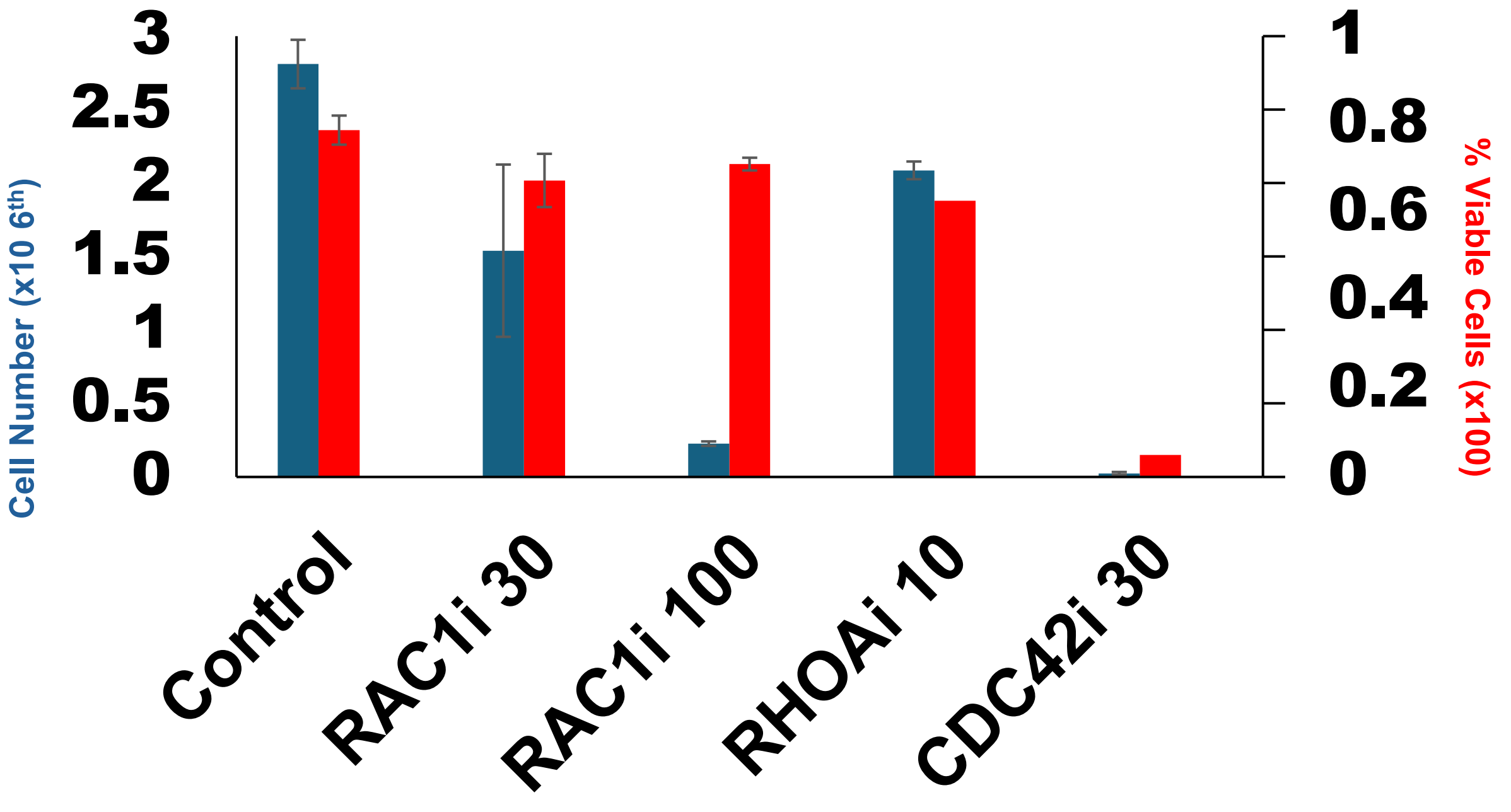
3
2.5
2
1.5
1
0.5
0
Cell Number (x10 6th)
1
0.8
0.6
0.4
0.2
0
% Viable Cells (x100)
Control
RAC1i 30
RAC1i 100
RHOAi 10
CDC42i 30

Figure S6

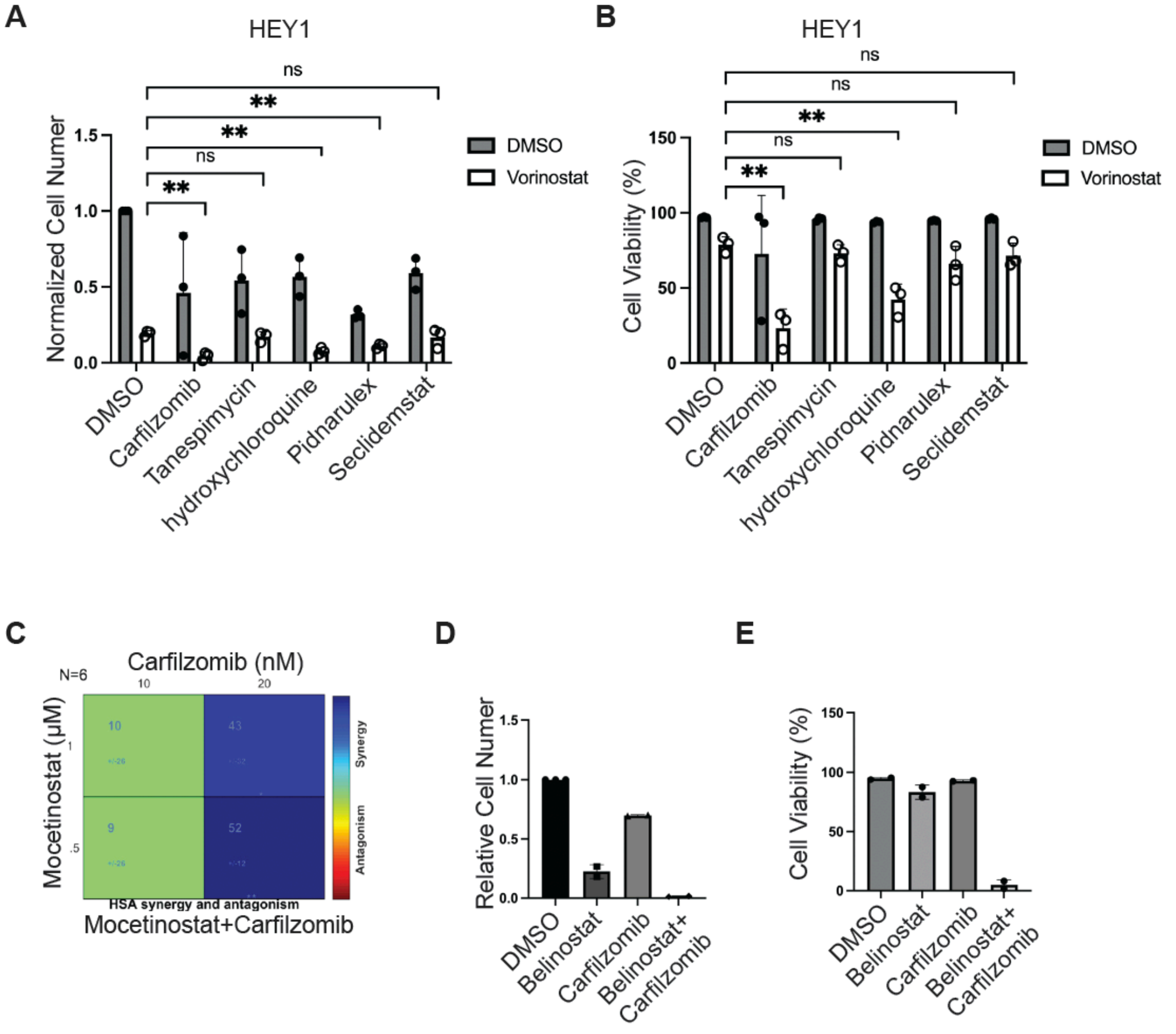
A
HEY1
ns
**
**
ns
**
Normalized Cell Numer
1.5
1.0
0.5
0.0
DMSO
Vorinostat
DMSO
Carfilzomib
Tanespimycin
hydroxychloroquine
Pidnarulex
Seclidemstat
B
HEY1
ns
ns
**
ns
**
Cell Viability (%)
150
100
50
0
DMSO
Vorinostat
DMSO
Carfilzomib
Tanespimycin
hydroxychloroquine
Pidnarulex
Seclidemstat
C
Carfilzomib (nM)
N=6
10
20
Mocetinostat (µM)
1
.5
10
43
9
52
Synergy
Antagonism
HSA synergy and antagonism
Mocetinostat+Carfilzomib
D
Relative Cell Numer
1.5
1.0
0.5
0.0
DMSO
Belinostat
Carfilzomib
Belinostat+
Carfilzomib
E
Cell Viability (%)
150
100
50
0
DMSO
Belinostat
Carfilzomib
Belinostat+
Carfilzomib
