# Supplementary material for: Targeting HDAC and proteostasis to eradicate quiescent cancer cells
Source: Cancer Gene Ther. 2026 Aug 13;33(9):1053–68. doi: 10.1038/s41417-026-01067-y (PMC13518249; doi:10.1038/s41417-026-01067-y)
Supplement: Supplementary file 2 [file 41417_2026_1067_MOESM1_ESM.docx]

**Supplemental Figure Legends**

**Figure S1 Validation of MBD3 and CHD4 siRNA and shRNA efficiency.** A. RT-qPCR of CHD4 and MBD3 in shRNA-transduced PT412 cells. B. Western blotting of CHD4 and MBD3 in shRNA stably transduced PT412 cells. C. RT-qPCR of CHD4 and MBD3 in siRNA-transfected PT412 cells. D. Western blotting of CHD4 and MBD3 in siRNA-transfected PT412 cells. E. Normalized cell counts of biologic replicates of PT412 cells transfected with the indicated siRNAs. F. Percent cell viability of PT412 cells transfected with the indicated siRNAs as measured by PI/Annexin-V flow cytometry.

**Figure S2 Vorinostat reversibly inhibited cell growth.** Incucyte real-time cell proliferation analysis, as measured by cellular confluency, of PT412 and Kuramochi cells treated with (1) DMSO, (2) vorinostat 72h followed by drug washout and treatment with DMSO (vorinostat→DMSO), or (3) vorinostat 72h followed by drug washout and the re-treatment with vorinostat (vorinostat→vorinostat).

**Figure S3 Violin plot for cell cycle marker expression**. Violin plot of protein expression of 28 cell cycle-related markers in control PT340 cells, PT340 cells grown under conditions of serum starvation (serum free), and cells treated with Vorinostat (SAHA). Expression level was measured via interative immunofluorescence as previously described ^32^.

**Figure S4 PCA analysis for RNA sequencing**. Principle component analysis plots of RNA sequencing results for three replicates of PT340 and PT412 cells treated with DMSO (CS) or vorinostat (TS). Results demonstrate cells clearly separate by treatment group, indicating a strong treatment effect.

**Figure S5 Impact of RHO/RAC/CDC42 pathway inhibitor on quiescence**. Total cell number and percentage of viable PT340 cells treated with the indicated doses of a RACi inhibitor (RAC1i; NSC 23766), a CDC42 inhibitor (CDC42i; ML-141), and a RHOA inhibitor (RHOAi; Rhosin hydrochloride). Numbers indicate doses in µM.

**Figure S6. Synergistic effect of HDACi and drugs with impact proteostasis.** A and B. Normalized cell number and percent viable cells in HEY1 cells treated with the indicated drugs in combination with DMSO (control) or vorinostat. C. HSA synergy heat map for Mocetinostat and carfilzomib in PT340 cells. D and E. Normalized cell number and viable percentage of OVSAHO cells treated with the indicated drug.
